# The *Bacillus anthracis* class Ib ribonucleotide reductase subunit NrdF intrinsically selects manganese over iron

**DOI:** 10.1007/s00775-020-01782-3

**Published:** 2020-04-15

**Authors:** Kristīne Grāve, Julia J. Griese, Gustav Berggren, Matthew D. Bennett, Martin Högbom

**Affiliations:** 1 Department of Biochemistry and Biophysics, Stockholm University, Svante Arrhenius väg 16C, 10691 Stockholm, Sweden; 2 Department of Cell and Molecular Biology, Uppsala University, BMC, Box 596, 75124 Uppsala, Sweden; 3 Department of Chemistry, Ångström Laboratory, Uppsala University, Lägerhyddsvägen 1, 75120 Uppsala, Sweden

**Keywords:** Metalloprotein, Metal selectivity, Quantitative X-ray anomalous dispersion, Ferritin superfamily

## Abstract

**Abstract:**

Correct protein metallation in the complex mixture of the cell is a prerequisite for metalloprotein function. While some metals, such as Cu, are commonly chaperoned, specificity towards metals earlier in the Irving–Williams series is achieved through other means, the determinants of which are poorly understood. The dimetal carboxylate family of proteins provides an intriguing example, as different proteins, while sharing a common fold and the same 4-carboxylate 2-histidine coordination sphere, are known to require either a Fe/Fe, Mn/Fe or Mn/Mn cofactor for function. We previously showed that the R2lox proteins from this family spontaneously assemble the heterodinuclear Mn/Fe cofactor. Here we show that the class Ib ribonucleotide reductase R2 protein from *Bacillus anthracis* spontaneously assembles a Mn/Mn cofactor in vitro*,* under both aerobic and anoxic conditions, when the metal-free protein is subjected to incubation with Mn^II^ and Fe^II^ in equal concentrations. This observation provides an example of a protein scaffold intrinsically predisposed to defy the Irving–Williams series and supports the assumption that the Mn/Mn cofactor is the biologically relevant cofactor in vivo. Substitution of a second coordination sphere residue changes the spontaneous metallation of the protein to predominantly form a heterodinuclear Mn/Fe cofactor under aerobic conditions and a Mn/Mn metal center under anoxic conditions. Together, the results describe the intrinsic metal specificity of class Ib RNR and provide insight into control mechanisms for protein metallation.

**Graphical Abstract:**

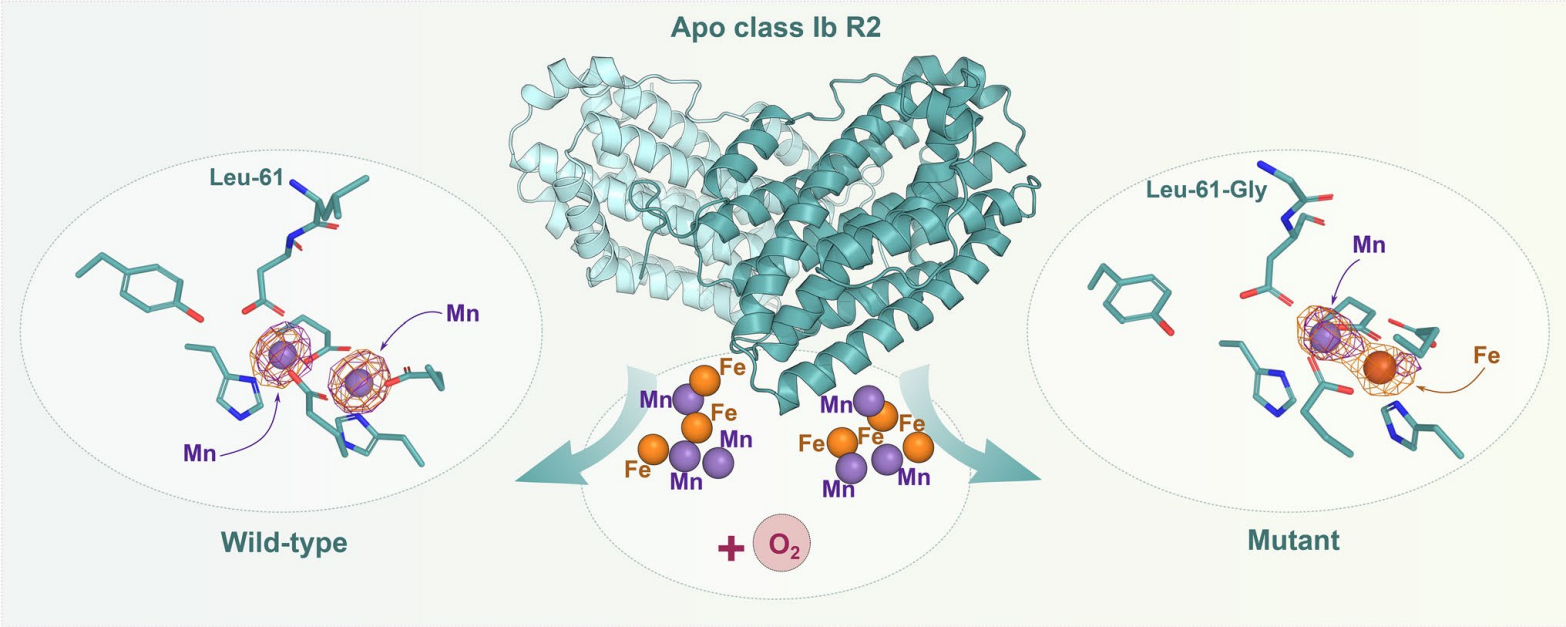

**Electronic supplementary material:**

The online version of this article (10.1007/s00775-020-01782-3) contains supplementary material, which is available to authorized users.

## Introduction

Achieving correct metallation in the complex environment of the cell is of paramount importance for the function of metalloproteins. While some systems compartmentalize metalloproteins with their required metals or employ specialized metallo-chaperones, divalent metal ion affinities to protein ligands are commonly described by the Irving–Williams series (Mn^II^ < Fe^II^ < Co^II^ < Ni^II^ < Cu^II^ > Zn^II^) [1, 2]. This order describes relative metal–ligand complex stability and is often an indirect indication of metal–ligand affinity. Cells tend to maintain the availabilities of metal ions inverse to the Irving–Williams series [3]. Mn and Fe represent an interesting case: both prefer similar coordination environments and bind weakly to their protein ligands [1]. During recent years it has been shown that protein scaffolds of R2-like ligand-binding oxidases (R2lox) and the radical-generating subunit in class Ic ribonucleotide reductases (R2c), both belonging to the ferritin superfamily, directly influence metal specificity by selectively forming a heterodinuclear Mn/Fe cofactor instead of an Fe/Fe metal center, apparently defying the Irving–Williams series [4, 5]. The protein scaffold can potentially control metallation by both steric effects and by influencing reactivity [4, 6].

**Fig. 1:**
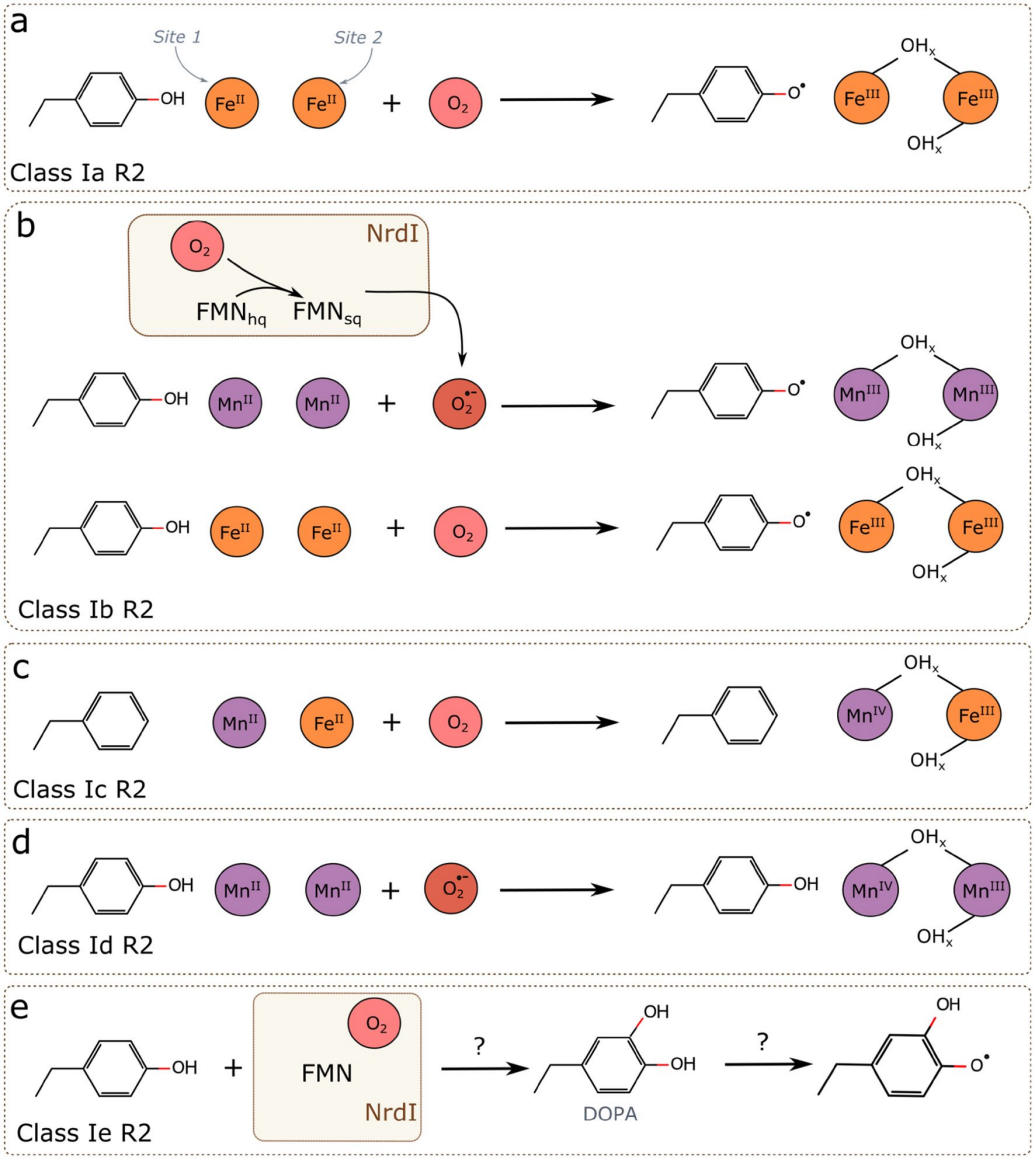
Schematic representation of different types of radicals in class I RNR R2 proteins together with simplified radical generation mechanisms. **a** Di-iron class Ia R2 (Refs. [13–15]). **b** Class Ib R2 can generate a tyrosyl radical in two ways: when a di-manganese form of the R2 reacts with superoxide, generated by NrdI protein; and when a di-iron form of the R2 reacts with molecular oxygen (Ref. [16]). **c** Mixed metallo-cofactor of class Ic R2 (Refs. [17–19]). **d** Di-manganese metallo-cofactor of class Id R2 (Refs. [10, 20, 21]. **e** Metal-free class Ie R2 (Refs. [11, 12]). *DOPA* 3,4-dihydroxyphenylalanine

Ribonucleotide reductase (RNR) is a key enzyme in all domains of life, utilizing radical chemistry for the synthesis of deoxyribonucleotides by substitution of the 2′ OH-group of a ribose 5′phosphate with a hydrogen [7]. RNRs are currently divided into three classes (I–III) based on the oxygen dependence, quaternary structure, and type of cofactor, essential for the radical generation (reviewed in [8, 9]). The strictly aerobic class I RNRs are comprised of two homodimeric subunits: a large catalytic subunit R1, where ribonucleotide reduction takes place, and a small radical-generating subunit R2. Allosteric regulation is predominantly performed in the R1 subunit but is also identified in some R2 proteins [10]. Class I R2 proteins are further subdivided into five subgroups (R2a–R2e), based on the radical species and/or metallo-cofactor employed for radical generation (Fig. 1). With the exception of a recently discovered metal-free class of R2 proteins (denoted R2e) [11, 12], R2 proteins house a dinuclear metallo-cofactor, utilized for radical generation. R2a proteins utilize an oxygen-activated di-ferrous metal site to generate a stable Fe^III^/Fe^III^-tyrosyl radical (Y**·**) cofactor [13–15], whereas R2b proteins generate a Mn^III^/Mn^III^-Y**·** [16]. In R2c and R2d subclasses, on the other hand, the radical equivalent is housed on the metal cofactor itself. R2c proteins generate a Mn^IV^/Fe^III^ cofactor [17–19] and the recently discovered class R2d—a Mn^IV^/Mn^III^ cofactor [10, 20, 21]. These dimetal centers reside in a ferritin-like scaffold, and are coordinated by two histidine residues and four carboxylate ligands. The radical species, generated in the R2 subunit, is subsequently transferred to the catalytic subunit R1 for the formation of a cysteine thiyl radical, which, in turn, initiates ribonucleotide reduction [7].

A related R2lox protein group was discovered based on sequence similarity to R2c proteins [22]. The R2lox proteins that have been structurally characterized to date were shown to contain a Mn/Fe heterodinuclear metallo-cofactor, similar to R2c enzymes, coordinated by four glutamate and two histidine residues, however they are not involved in ribonucleotide reduction [4, 22]. In contrast to R2c, R2lox performs two-electron redox chemistry, forming a tyrosine–valine ether cross-link in the vicinity of its metal binding site during cofactor maturation [4, 23]. Cofactor maturation in R2c and R2lox proteins appears to follow a common pathway [4, 24–29], but the assembly pathways of the metal sites differ [4, 5, 30]. The physiological role of R2lox proteins is yet to be discovered.

The di-ferrous metallo-cofactor of R2a is capable of reducing molecular oxygen for the generation of a stable Y**·** [13–15]. R2b enzymes are also capable of Y**·** generation using a di-ferrous metallo-cofactor similar to R2a, but are less efficient [31]. Instead, Mn^II^ was proposed to be the relevant metal ion in vivo for the R2b enzymes [32–35]. In contrast to the di-Fe^II^ cofactor, di-Mn^II^ is unreactive towards O_2_ directly and requires a superoxide species, provided by an FMN coenzyme-containing activase, NrdI [16]. R2d proteins, which also utilize Mn^II^, scavenge the superoxide from solution and do not require NrdI [20, 21]. Heterodinuclear R2c and R2lox proteins, in turn, are capable of reducing O_2_ directly via a proposed Mn^IV^/Fe^IV^ intermediate without any auxiliary factors [4, 24–28].

Recently, Kutin et al. showed that a second coordination sphere mutation in R2lox from *Geobacillus kaustophilus* (*Gk*), which normally assembles a heterodinuclear Mn/Fe cofactor, affects both metallo-cofactor composition and its maturation pathway during oxygen activation, resulting in a cofactor with a more R2c-like electronic structure [29]. This was achieved by substituting a highly conserved Gly68 residue in R2lox, directly preceding the N-terminal metal-coordinating carboxylate, for a leucine side chain. There is no strict conservation of the corresponding residue in R2 proteins, but it is generally hydrophobic. In R2b proteins, however, a leucine is commonly found in this position, as is the case in *Bacillus anthracis* (*Ba*) R2b [29, 36].

Here, we use quantitative X-ray anomalous dispersion (Q-XAD) [37] to show that the *Ba*R2b protein scaffold has an intrinsic metal specificity allowing it to select Mn over Fe in both metal binding positions. This observation supports the assignment of di-manganese as the biologically relevant cofactor in vivo*.*

To start to elucidate the mechanism behind the remarkable specificity for manganese over iron in *Ba*R2b, we set out to investigate if a reverse R2lox-like Leu61-Gly substitution in *Ba*R2b affects metallo-cofactor formation. Interestingly, the Leu61–Gly mutation allows *Ba*R2b to assemble an oxygen-reactive Mn/Fe heterodinuclear cofactor, similar to R2lox and R2c. Mechanisms that could contribute to this behavior and the manganese specificity of the wild-type protein are discussed.

## Materials and methods

### Site-directed mutagenesis

A L61G substitution was prepared using a QuikChange Lightning (Agilent) site-directed mutagenesis kit and the following primers (5′–3′) with an introduced mutation site underlined:

L61G_for: GTGTATTAGGTGGTTTAACACTTgccGATACGAAACAAGGCGGC

L61G_rev: CCGCCTTGTTTCGTATCgccAAGTGTTAAACCACCTAATACACGC

Wild-type (WT) *B. anthracis nrdF* coding sequence (UniProt: Q81TB4) cloned into pET22b (Novagen) vector [38] was used as a PCR template. The presence of the desired mutation was confirmed by sequencing.

### Protein expression and purification

Non-His-tagged WT and L61G *Ba*R2b variant were expressed and purified in identical conditions, as described before for the WT protein [38] with an additional de-metallation step [39]. Briefly, proteins were heterologously expressed in aerobically cultured *Escherichia coli* Rosetta 2 (DE3) (Novagen) in LB medium in the presence of 25 μg/mL chloramphenicol and 50 μg/mL carbenicillin. Cells were induced with 1 mM isopropyl-*b*-d-thiogalactopyranoside and cultured for 4 h at 37 °C before harvesting by centrifugation. Cells were disrupted using the X-press (BIOX) and extracted in 50 mM Tris–HCl (pH 7.5), 20 mM MgCl_2_, 20 mM DTT, and protease inhibitor mixture tablets (Roche Life Science). Supernatant was cleared by centrifugation. Both WT and L61G variant *Ba*R2b were purified by ammonium sulfate precipitation followed by hydrophobic affinity chromatography and anion exchange chromatography. To remove any bound metal ions, proteins were incubated with 10 mM EDTA and 2.5 mM sodium dithionite on ice for 1.5 h and re-chromatographed on a HiLoad Superdex 200 16/60 pg (GE Healthcare) in 20 mM Tris–HCl pH 7.5 and 100 mM KCl. *Ba*R2b protein-containing fractions were pooled, assessed for purity by SDS-PAGE, concentrated to 15–30 mg/mL, flash-cooled in liquid N_2_ and stored at − 80 °C until further use. The metal content of the purified proteins was assessed by TXRF (described below).

### Total-reflection X-ray fluorescence spectrometry (TXRF)

Metal content of the apo-protein preparations were quantified using TXRF analysis on a Bruker PicoFox instrument [40]. A gallium standard (Sigma) was added to duplicate samples (v/v 1:1) prior to the measurements. TXRF spectra were analyzed using the routines provided with the spectrometer.

### Crystallization and crystallographic data collection

WT metal-free *Ba*R2b was crystallized by hanging drop vapor diffusion experiments as described before [39]. Metal-free L61G variant crystallized in identical conditions.

To obtain metal-bound crystals, apo-protein crystals were soaked in mother liquor containing excessive and equal amounts of freshly prepared Mn^II^ and Fe^II^ salts. Crystal soaks were performed (1) in presence of oxygen and (2) in anoxic conditions:

(1) Apoprotein crystals (WT and L61G) were soaked for 20–30 min in 2–3 µL air-saturated cryoprotective mother liquor drop additionally containing 25% (v/v) glycerol and 5 mM each of MnCl_2_ and (NH_4_)_2_Fe(SO_4_)_2_.

(2) Apoprotein crystals (WT and L61G) were soaked anoxically for 45–60 min in 1 mL of cryoprotective mother liquor additionally containing 5 mM each of MnCl_2_ and (NH_4_)_2_Fe(SO_4_)_2_, 30 mM sodium dithionite, 0.5 mM phenosafranin and 25% (v/v) glycerol. Crystals were flash cooled in liquid nitrogen prior to data collection.

Data were collected at 100 K at X06SA/SLS beamline (Villigen, Switzerland), BL14.2/BESSY (Helmholtz Center Berlin, Germany) and ID23-1/ESRF (Grenoble, France). For the purpose of metal quantification, data collection proceeded in the order Mn K-edge (6559 or 6589 eV, 1.890 Å wavelength)—Fe K-edge (7132 or 7162 eV, 1.738 Å wavelength) on the same crystal. High-resolution native data were collected after anomalous datasets.

### Structure determination and refinement

Native data were processed with XDS [41] or DIALS [42], merged and scaled with AIMLESS [43]. High-resolution cut-off was decided based on CC_1/2_ and *I*/*σ* (*I*) in the highest resolution shell. Structures were solved using molecular replacement using phases from a monomer of *B. anthracis* R2b structure (PDB id: 6QO9) [39] with all ligands removed using Phaser (Phenix suite) software [44, 45]. The models were iteratively rebuilt using Coot [46] and refined using phenix.refine [47]. Refinement generally included bulk solvent corrections, individual atomic coordinate and occupancy refinement for alternate conformations. Due to the sufficiently high resolution of the data for aerobically prepared WT *Ba*R2b dataset (1.35 Å) anisotropic *B* factors were refined (including metal ions). Solvent molecules were added with phenix.refine and manually. Metal–ligand bonds were restrained in all structures, except the aerobically metal-loaded L61G variant. Due to mixed oxidation states and lower metal occupancy in site 1 in this structure (discussed further in “Results” section), metal–ligand coordination was assigned manually. Structures were validated using MolProbity [48]. Refinement and model quality statistics are presented in Table 1. Structure figures were prepared in PyMOL [49].

**Table 1:** Data collection and refinement statistics

	Aerobic WT	Anoxic WT	Apo L61G	Aerobic L61G	Anoxic L61G
PDB code	6TQV	6TQW	6TQX	6TQY	6TQZ
Data collection statistics					
Wavelength (Å)	1.04	1.04	0.97	0.92	0.92
Resolution range (Å)	54.35–1.35 (1.39–1.35)	45.7–1.55 (1.58–1.55)	54.49–2.05 (2.12–2.05)	46.02–2.10 (2.17–2.10)	41.56–1.77 (1.83–1.77)
Space group	*P*2_1_	*P*2_1_	*P*2_1_	*P*2_1_	*P*2_1_
Unit cell dimensions *a*, *b*, *c* (Å)	57.18 60.3 95.8	57.50 61.28 95.37	57.34 60.49 95.60	57.02 60.12 96.05	57.25 60.49 95.99
Unique angle: *β* (°)	106.47	106.61	106.62	106.52	106.53
Unique reflections	131,966 (11,538)	88,660 (8520)	38,704 (3903)	36,123 (3585)	60,553 (5901)
Multiplicity	3.5 (2.9)	3.8 (3.7)	3.0 (3.0)	3.8 (3.9)	3.8 (3.7)
Completeness (%)	96.05 (84.30)	95.98 (92.66)	98.00 (98.78)	98.21 (97.68)	98.06 (95.84)
Mean *I*/sigma (*I*)	12.3 (1.4)	16.7 (5.3)	6.5 (2.2)	9.4 (3.0)	14.0(2.4)
Wilson B-factor (Å^2^)	13.79	15.22	23.19	25.46	22.99
R-merge	0.036 (0.589)	0.035 (0.180)	0.079 (0.390)	0.089 (0.383)	0.054 (0.493)
R meas	0.049 (0.803)	0.047 (0.241)	0.110 (0.543)	0.104 (0.444)	0.063 (0.575)
R pim (*I*)	0.032 (0.548)	0.031 (0.159)	0.077 (0.377)	0.0523 (0.221)	0.0319 (0.292)
CC_1/2_	0.965 (0.629)	0.993 (0.975)	0.997 (0.902)	0.997 (0.928)	0.999 (0.868)
Refinement statistics					
Reflections used in refinement	131,716 (11,507)	88,562 (8511)	38,570 (3887)	36,024 (3577)	60,442 (5891)
Reflections used for R-free	6664 (561)	4554 (460)	1889 (200)	1799 (179)	3018 (295)
R-work	0.15 (0.23)	0.16 (0.19)	0.17 (0.22)	0.17 (0.18)	0.17 (0.23)
R-free	0.17 (0.27)	0.18 (0.22)	0.21 (0.28)	0.22 (0.24)	0.21 (0.28)
Protein residues	571	576	570	572	573
Number of non-hydrogen atoms	5061	5155	4914	4864	4967
Number of water molecules	353	416	250	208	278
Number of metal ions	4	4	0	4	4
RMS, bonds (Å)	0.010	0.009	0.012	0.012	0.011
RMS, angles (°)	1.09	1.03	1.10	1.06	0.94
Ramachandran favored/allowed/outliers (%)	99.29/0.71/0	99.13/0.87/0	98.93/1.07/0	98.41/1.59/0	98.12/0.88/0
Rotamer outliers (%)	0.99	0.20	0.60	1.61	0.20
Clashscore	1.17	1.06	1.83	2.49	0.97
Average B-factor (Å^2^)	20.49	19.89	25.70	29.58	27.96
Macromolecules	19.61	19.02	25.34	29.58	27.47
Ligands	41.72	33.87	44.98	47.71	46.75
Metal ions	12.07	11.84	–	44.25	23.19
Solvent	30.40	28.75	30.05	32.48	34.34

Values in parentheses are for the highest resolution shell

### Analysis of anomalous diffraction data

Analysis of anomalous diffraction data and metal quantification were done essentially as described in ref.[37]. Data were integrated with DIALS [42]. Each dataset was individually scaled and merged with AIMLESS [43] with anomalous pairs separated. Additionally, the Fe and Mn datasets from each crystal were placed on a common scale with XSCALE [41] and merged with AIMLESS with anomalous pairs separated. All data was processed over the same resolution range (46–3 Å). Data quality statistics is presented in Supplementary Table 2. Both relatively scaled and unscaled Mn- and Fe-edge datasets from the same crystal were analyzed as a part of data quality control procedure to exclude unwanted data bias caused by radiation damage. Anomalous difference maps were calculated with Phenix [45] using the anomalous structure factor amplitudes from the Mn- and Fe-edge datasets and phases from Mn-bound *Ba*R2b (PDB id: 6QO9) [39] with all ligands removed. Generated maps were examined in Coot [46]. To estimate the relative amounts of Fe and Mn in both metal sites of each protomer of the *Ba* NrdF homodimer, the intensities of the anomalous difference density peaks in spheres of 1.7–1.9 Å radius were integrated using MAPMAN [50]. The relative amounts of Fe and Mn in each site can be estimated from the integrated intensities at the Fe and Mn edges when taking into account the different contributions of both elements to the anomalous signal at the two wavelengths, if the total occupancy of each metal site is assumed to be 100%. The quantification results obtained from scaled and unscaled datasets were the same to within 10%.

## Results

Recently we reported several *B. anthracis* R2b crystal structures in complex with Mn, Fe and in the metal-free (apo) form [39]. To study metal selectivity and cofactor assembly from a mixed Mn^II^ and Fe^II^ pool in the presence and absence of O_2_, in the present study we describe apoprotein crystal soaking experiments, subjecting apo *Ba*R2b to a large excess of both Mn^II^ and Fe^II^ in aerobic and reducing anoxic conditions (in presence of 30 mM sodium dithionite). In addition, we have introduced an R2lox-like L61G mutation in the second coordination sphere in close proximity to the N-terminal metal binding site (site 1) and investigated its effect on the structure and protein metallation. The location-specific metal content of metal-loaded proteins was analyzed by Q-XAD [37]. To ensure that the protein was metal-free prior to cofactor reconstitution, WT and the variant were analyzed by TXRF. The content of Fe, Mn and other transition metals (Co, Ni, Cu and Zn) in the sample were negligible (≤ 0.1 metal per protein). The metal-free crystals of the variant protein were subjected to the same set of metal-loading experiments as the WT *Ba*R2b (see “Materials and methods” for details).

### Structure of Ba*R2b L61G*: The L61G mutation affects metal site geometry

**Fig. 2:**
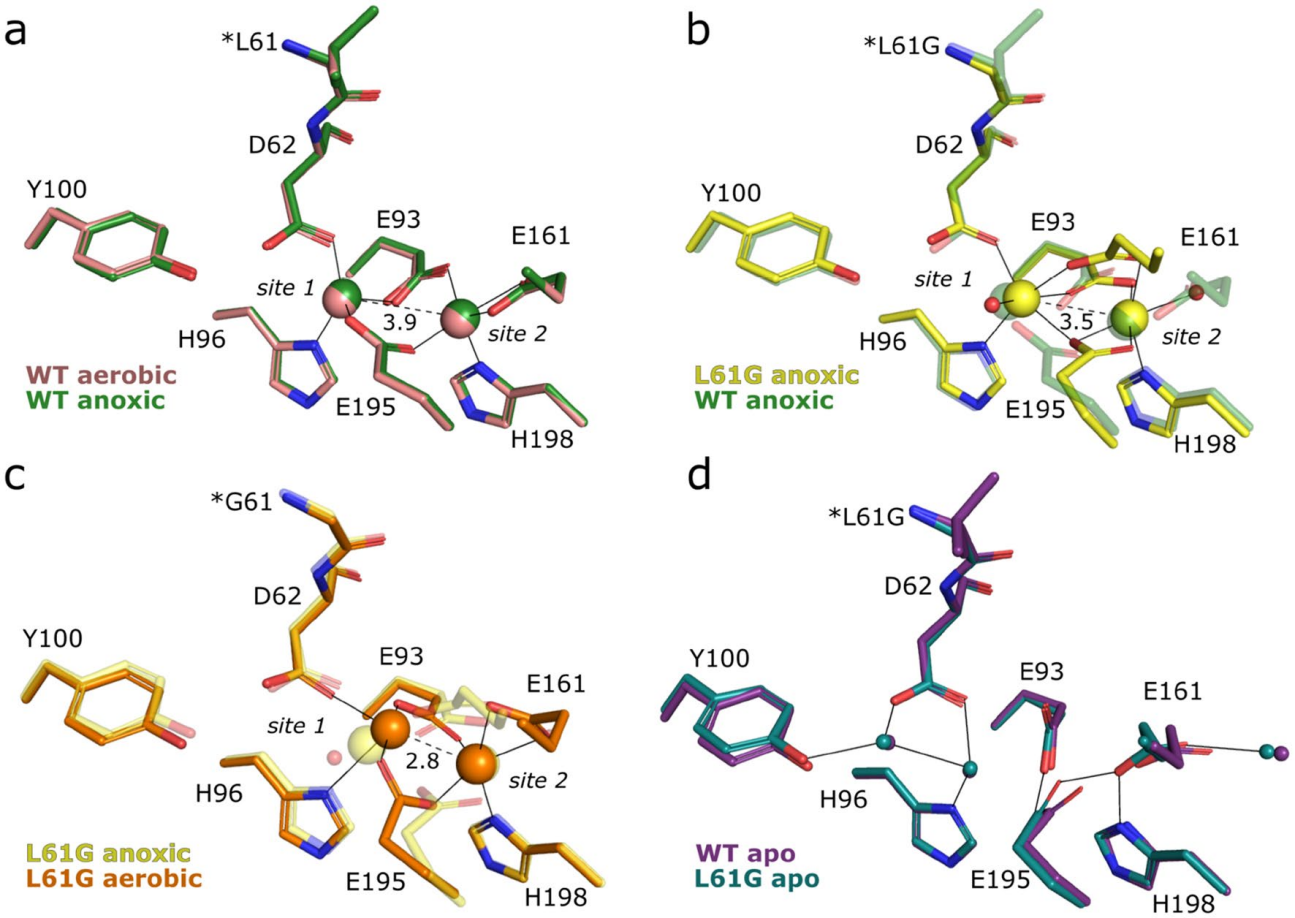
Coordination environment of WT and L61G variant BaR2b metal sites in crystals prepared without oxygen (**a**, **b**) and aerobically (**c**, **d**). The site of the mutation is marked with an asterisk. **a** Metal site of WT BaR2b crystal soaked with Mn^II^ and Fe^II^ in anoxic (green) and aerobic conditions (pink). **b** Overlay of the L61G (yellow) and WT R2b (transparent green, as in **a**) metal sites from crystals soaked with equal amount of Mn^II^ and Fe^II^ in anoxic conditions. **c** Overlay of the anaerobically prepared L61G (transparent yellow, as in **b**) and L61G R2b (orange), prepared in aerobic conditions. Crystals were soaked with equal amount of Mn^II^ and Fe^II^. **d** Overlay of the metal sites in WT (purple) and L61G (blue) apo R2b. Metal ions are shown as large spheres, solvent molecules—as small spheres. Solid lines indicate hydrogen and metal coordinations, while dashed lines indicate metal–metal distances (in Å)

Overall, in all structures reported in this study, the first 288 residues could be traced in the electron density, leaving the last 34 C-terminal residues disordered. The electron density is well-defined, except for a flexible poly-glutamate loop region E275–E278, as noted before [39]. X-ray data collection and structure refinement statistics are presented in Table 1. Crystal structures of the wild-type *Ba*R2b, prepared with Mn and Fe in aerobic or anoxic conditions, are within experimental error identical to previously reported *Ba*R2b in the reduced metallo-cofactor state (Fig. 2a) [39].

Leu61 is one of the residues forming a hydrophobic pocket around the radical-harboring Tyr100, and directly precedes the metal-coordinating Asp62 (Fig. 2). The substitution of Leu61 with Gly allows the Phe165 side chain, located on αE, to adopt an alternative conformation, occupying the vacant space (Supplementary Fig. 2a), creating a larger hydrophobic cavity around the metal center. Interestingly, the distorted part of the αE (residue stretch 159–168), adopting a π/3_10_-helical conformation in all L61G structures, has an increased B-factor for the Cα atoms, suggesting that the mutation has a destabilizing effect in this region (Supplementary Fig. 2). Additionally, the mutation renders a larger surface-connected cavity between helices αB (residues 46–76) and αD (residues 125–140), gated by Met1 and Glu127 (Supplementary Fig. 1b, c).

The mutation induced significant rearrangements in the metal coordination sphere, as compared to WT protein (Fig. 2b, c). In the crystal structure anaerobically prepared with Mn and Fe both metal sites refine to nearly full-occupancies in both chains of the homodimer (85–90% in site 1 and 100%—in site 2). Based on anomalous dispersion data (discussed further below), Mn^II^ was modelled in both sites. In contrast to WT *Ba*R2b, each Mn^II^ is hexacoordinate. Interestingly, the metal site resembles the reduced structure of *E. coli* (*Ec*) di-Mn^II^ R2b metallo-cofactors with the unusually positioned Glu161 (Glu158 in *Ec*) and two solvent molecules coordinating one Mn^II^ each [51]. Specifically, both metal sites in *Ba*R2b are bridged by three glutamate ligands—Glu93, Glu161 and Glu195. A small population of alternative Glu161 conformation is observed in both chains of the homodimer, both coordinating the Mn ion in site 2 in a monodentate fashion and, most likely, replacing the bound solvent molecule (Supplementary Fig. 3a). A solvent molecule, His96 and Asp62 additionally coordinate to site 1. In turn, His198 and a solvent molecule coordinate the Mn ion in site 2 (Fig. 2b).

In the L61G crystal structure prepared with Mn and Fe in presence of O_2_ the metal site is generally more disordered and the metal site occupancies refine to 54–58% in site 1 and 72–80% in site 2. Based on anomalous dispersion data (discussed further below) Mn^II^ was modelled in site 1 and Fe^II^ in site 2. The exact metal coordination geometry could not be unambiguously established due to low occupancy of the metal ions and metal type inhomogeneity. In addition, the metallo-cofactor of the aerobically prepared L61G *Ba*R2b displays features of an oxidized state: metal–metal distance is decreased from 3.5 Å in anaerobically prepared L61G variant to 2.8–3.0 Å and the *F*_o_–*F*_c_ positive difference density at the metal site indicates the presence of a bridging species *trans* relative to the metal-coordinating histidine side chains (Supplementary Fig. 4a, b). Likely, the metallo-cofactor in this structure represents a mixture of reduced and oxidized states due to a combination of partial oxygen activation and photo-reduction during data collection. Metal site 1 is coordinated by His96 and Asp62 in a monodentate fashion, whereas site 2 by Glu161 in a bidentate fashion. Low occupancy of the metal in site 1 renders metal coordination by Glu195 and Glu193 ambiguous; however, both side chains are likely bridging both metal ions (Fig. 2c; Supplementary Fig. 4a, b). The structure of the metal-free L61G variant closely resembles the WT *Ba*R2b (Fig. 2d). The structure of the putative superoxide passage [51] to the metal in site 2 was not affected by the mutation.

### Wild-type *B. anthracis R2b* assembles a Mn/Mn metallo-cofactor

**Fig. 3:**
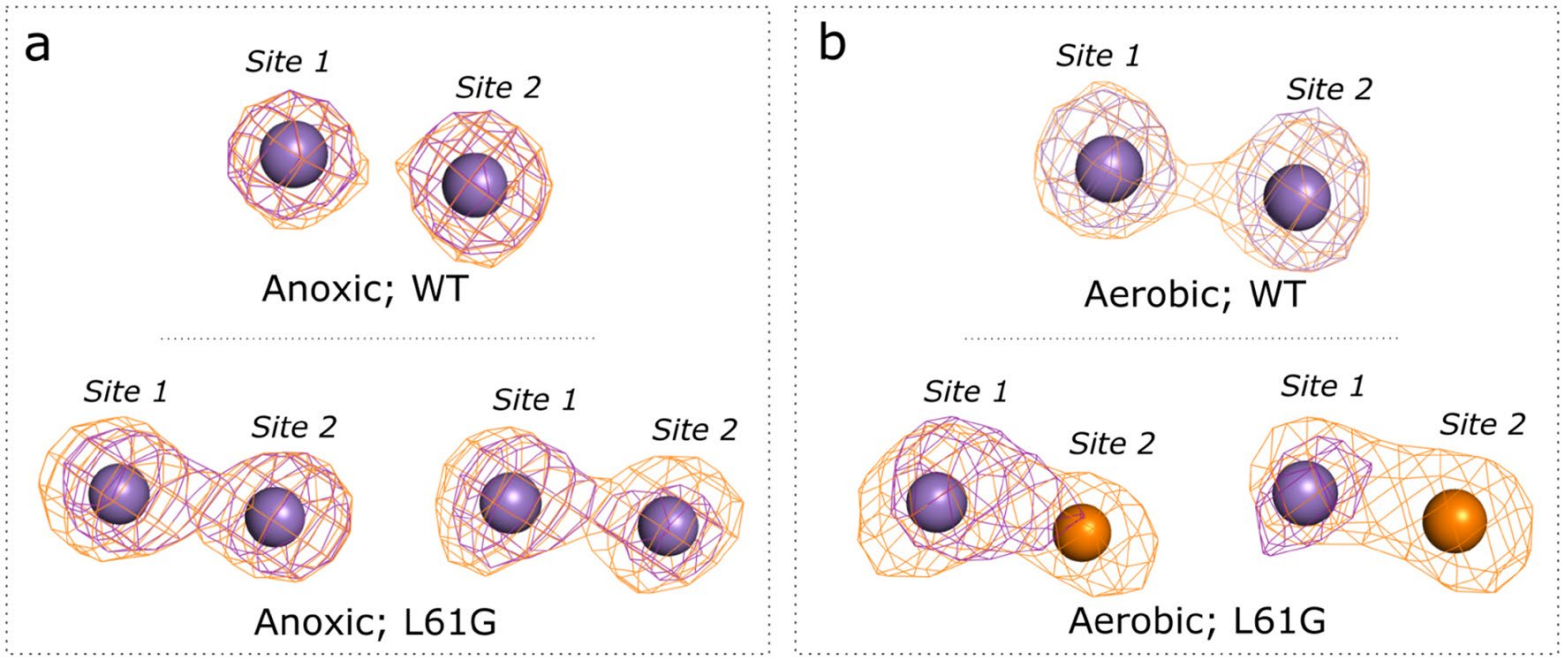
Representative anomalous difference density at the Mn (purple) and Fe (orange) edges from WT and L61G variant apoprotein crystals, soaked with Mn^II^ and Fe^II^ in **a** anoxic and **b** aerobic conditions. Difference density maps for the metal sites in WT BaR2b protein and anaerobically reconstituted L61G variant are contoured at 5σ; for the aerobically reconstituted L61G cofactor—at 4σ. At the Fe edge, both Mn and Fe display an anomalous signal. Quantification of metal content in each position is done via peak integration of the respective anomalous difference maps (Q-XAD [37])

Site-specific metal quantification by X-ray anomalous dispersion on WT *Ba*R2b revealed that the two sites of the metal center predominantly bind Mn irrespective of the presence or absence of O_2_ in the experimental setup. (Fig. 3; Supplementary Table 1). Notably, both metal binding sites show equivalent selectivity for Mn and thus diverge from what would be predicted by the Irving–Williams series.

### Under anaerobic conditions both metal sites of the metallo-cofactor in the *L61G* variant are more permissive to Fe binding

Site-specific metal quantification by X-ray anomalous dispersion in anaerobically prepared crystals revealed that both metal sites still display a propensity to mainly bind Mn^II^. However, the variant appears to be slightly more permissive for Fe^II^ binding in both positions, compared to the wild-type (Fig. 3, Supplementary Table 1).

### In the presence of oxygen, the *L61G* variant accumulates a heterodinuclear Mn/Fe cofactor

**Fig. 4:**
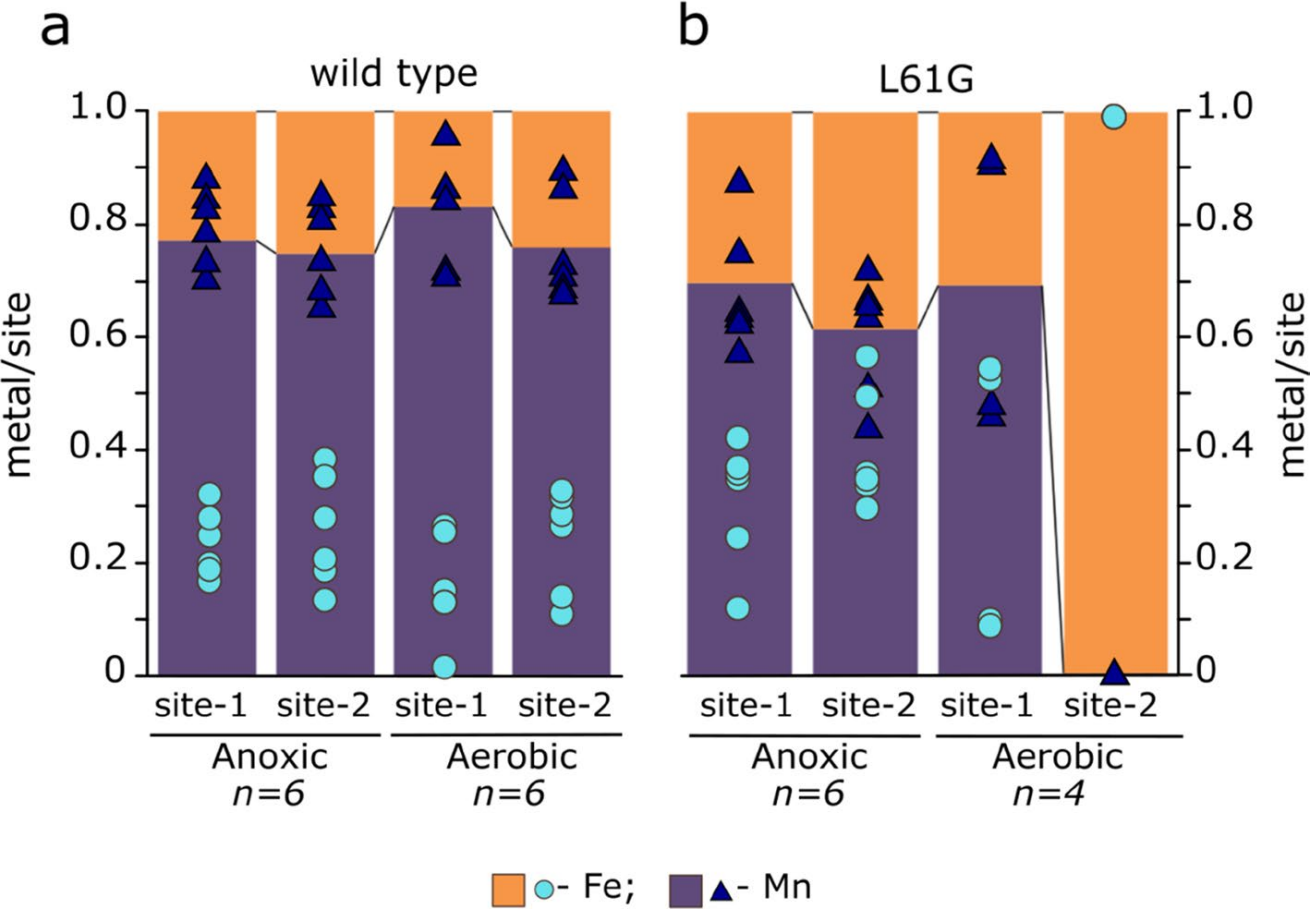
Relative amounts of Mn and Fe in the two metal sites in **a** WT and **b** L61G variant BaR2b obtained when reconstituting the cofactor in absence and presence of oxygen. The relative amounts (shown in orange for Fe and purple for Mn) are derived from the integrated intensity of the anomalous difference density peaks at the Fe and Mn absorption edges. Individual measurements (*n* number of metal sites) are depicted as cyan circles and blue triangles. The values for each measurement are given in Supplementary Table 1

The structure of the aerobically prepared L61G metallo-cofactor is more disordered, compared to the reduced cofactor or the wild-type protein, as can be deduced from the high B-factor values of the metal ions (see Table 1) and elongated shape of the anomalous difference density (Fig. 3b). We presume that the observed variability and structural dynamics of the metal binding site reflects the ability of the heterodinuclear metallo-cofactor to react with O_2_. Q-XAD results show that site 1 remains mainly Mn-specific in presence of O_2_. In contrast, site 2 now exclusively binds Fe (Fig. 4a, b).

## Discussion

The crystallographic data presented here shows that R2b proteins are capable of intrinsic selection of Mn over Fe, defying the Irving–Williams series, and provide a new example of a protein scaffold being able to regulate metal ion selectivity without assistance of any auxiliary factors. Unlike R2lox and R2c, wild-type *Ba*R2b is able to select Mn over Fe in both binding sites. The capability to exclude Fe appears central for a protein requiring a di-Mn cofactor for function. As Fe/Fe or Mn/Fe cofactors can react directly with molecular oxygen, they would become “trapped” in the protein in an oxidized state, rendering an inactive protein pool. The determinants for this remarkable specificity are not fully understood. However, recent experimental evidence suggested that a single amino acid exchange in the second metal coordination shell may change metallo-cofactor identity and its maturation pathway [29]. Given that site-specific metal quantification is required for this study, experiments were done using crystallized protein. Previous studies show that the in vivo metallation status and solution metal titrations of proteins from the ferritin superfamily generally agree well with what is observed in crystals [4, 22, 32, 33], suggesting that the *in crystallo* results are also relevant for the in vivo situation.

A Leu61 residue, directly preceding the site 1 metal-chelating Asp62 in *Ba*R2b, is conserved as glycine in the R2lox group that forms a heterodinuclear Mn/Fe site. Kutin et al. recently showed, that the substitution of this glycine side chain to a leucine had profound effects on metal selectivity in R2lox. Specifically, G68L R2lox became more permissive for Mn binding in site 2 in presence of oxygen [29]. Here we introduced a “reverse”, R2lox-like mutation L61G into the *Ba*R2b protein scaffold to investigate if this residue had similar effects in this scaffold.

The L61G substitution in *Ba*R2b resulted in significant side chain rearrangements in the metal-coordinating residues and secondary coordination sphere. Particularly the L61G mutation caused destabilization of the π/3_10_-helical segment of αE, which, in turn, allowed additional carboxylate ligand flexibility at the metal site and rendered both metal ions hexacoordinate, compared to four- and five-coordinate sites 1 and 2, respectively in the reduced *Ba*R2b structures (this work and ref. [39]). Interestingly, metal coordination number and geometry in the anaerobically reconstituted *Ba*R2b L61G variant highly resembles the metal coordination in Mn/Fe *Gk*R2lox, prepared anaerobically [4]. Glu161 (*Ba* numbering) bridges the metal ions similarly to the fatty acid bound to R2lox; the solvent molecule coordinating Mn in site 2 in *Ba*, on the other hand, is replaced by monodentate-coordinating Glu167 in *Gk*R2lox. Phe165, shown to be flexible in L61G *Ba*R2b variant, is an alanine in R2lox and lines the fatty acid binding cavity (Supplementary Fig. 6a). This alanine residue (Ala171 in *Gk*R2lox) is conserved in the R2lox group; in R2c and other canonical R2 proteins it is conserved as phenylalanine with some rare exceptions [36]. The conformational space created after L61G substitution in *Ba*R2b likely influences metal selectivity. Q-XAD measurements of anaerobically prepared samples showed that both metal sites became somewhat more permissive to Fe binding as compared to the WT protein (Fig. 4).

**Fig. 5:**
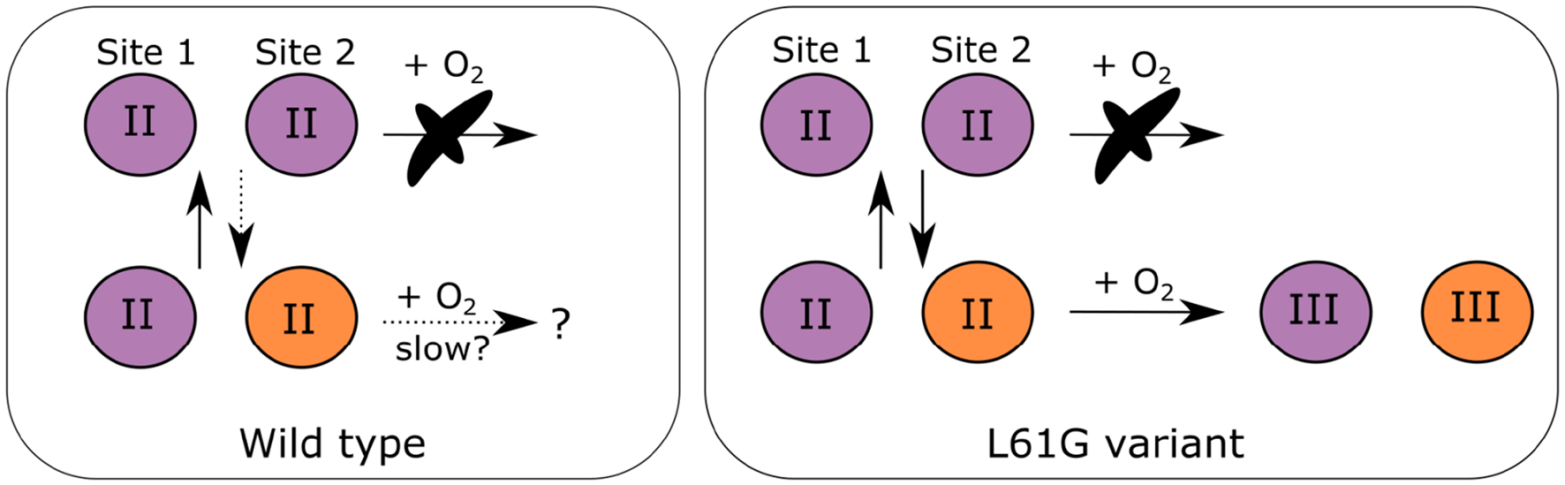
Schematic summary of metallation and cofactor activation by oxygen in (left panel) wild-type and L61G variant (right panel) *B. anthracis* R2b crystals. Purple spheres represent Mn and orange—Fe. Roman numerals represent metal ion oxidation states

Reconstitution of L61G apoprotein crystals with Mn and Fe in presence of O_2_, on the other hand, resulted in dramatic Fe enrichment in site 2, as in R2lox and R2c proteins [4, 5, 22, 30]. Thus, the variant protein was shown not only to assemble a Mn/Fe heterodinuclear metallo-cofactor, but also to reduce O_2_, as evident from the crystallographic data (Supplementary Fig. 4). The data suggest that both metal sites could in essence bind either Mn^II^ or Fe^II^; and, before the cofactor is activated by oxygen, bound metal ions may exchange with the labile pool of divalent metal ions available in solution. If Mn binds in both positions, neither the wild-type nor the L61G reacts with O_2_. However, it appears that once Mn^II^ is bound to site 1 and Fe^II^ to site 2, the metallo-cofactor in the L61G variant is able to react with O_2_ and trap the bound metals in the sites (Fig. 5)—something that is not observed to a significant degree in the wild-type protein. This observation is consistent with the effects induced by an “opposite” mutation in R2lox, where the G68L substitution alters the electronic structure of the metallo-cofactor, thus changing its redox potential [29]. Together these results suggest that the identity of the residue preceding the N-terminal metal ligand is a central determinant for metal selectivity and that this is achieved through modulating both metal coordination and cofactor reactivity in the R2 protein family.

## Electronic supplementary material

Below is the link to the electronic supplementary material.

Supplementary file1 (PDF 9805 kb)

## Abbreviations

FMN: Flavin mononucleotide
PDB: Protein data bank
Q-XAD: Quantitative X-ray anomalous dispersion
R1: Large subunit of ribonucleotide reductase, also called α
R2: Radical-generating subunit of ribonucleotide reductase, also called β
R2lox: R2-like ligand-binding oxidase
RNR: Ribonucleotide reductase
TXRF: Total-reflection X-ray fluorescence
WT: Wild-type

## References

[1] Cotruvo JA, Stubbe J (2012). Metallation and mismetallation of iron and manganese proteins in vitro and in vivo: the class I ribonucleotide reductases as a case study. Metallomics.

[2] Irving H, Williams RJP (1953) The stability of transition–metal complexes. J. Chem. Soc. (Resumed) 3192–3210

[3] Osman D, Martini MA, Foster AW (2019). Bacterial sensors define intracellular free energies for correct enzyme metalation. Nat Chem Biol.

[4] Griese JJ, Roos K, Cox N (2013). Direct observation of structurally encoded metal discrimination and ether bond formation in a heterodinuclear metalloprotein. Proc Natl Acad Sci USA.

[5] Dassama LMK, Krebs C, Bollinger JM (2013). Structural basis for assembly of the Mn^IV^/Fe^III^ cofactor in the class Ic ribonucleotide reductase from *Chlamydia trachomatis*. Biochemistry.

[6] Kisgeropoulos EC, Griese JJ, Smith ZR (2020). Key structural motifs balance metal binding and oxidative reactivity in a heterobimetallic Mn/Fe protein. J Am Chem Soc.

[7] Nordlund P, Reichard P (2006). Ribonucleotide reductases. Annu Rev Biochem.

[8] Hofer A, Crona M, Logan DT, Sjöberg B-M (2012). DNA building blocks: keeping control of manufacture. Crit Rev Biochem Mol Biol.

[9] Torrents E (2014). Ribonucleotide reductases: essential enzymes for bacterial life. Front Cell Infect Microbiol.

[10] Rozman Grinberg I, Lundin D, Hasan M (2018). Novel ATP-cone-driven allosteric regulation of ribonucleotide reductase via the radical-generating subunit. eLife.

[11] Srinivas V, Lebrette H, Lundin D (2018). Metal-free ribonucleotide reduction powered by a DOPA radical in Mycoplasma pathogens. Nature.

[12] Blaesi EJ, Palowitch GM, Hu K (2018). Metal-free class Ie ribonucleotide reductase from pathogens initiates catalysis with a tyrosine-derived dihydroxyphenylalanine radical. Proc Natl Acad Sci USA.

[13] Atkin CL, Thelander L, Reichard P, Lang G (1973). Iron and free radical in ribonucleotide reductase. Exchange of iron and Mossbauer spectroscopy of the protein B2 subunit of the *Escherichia coli* enzyme. J Biol Chem.

[14] Ehrenberg A, Reichard P (1972). Electron spin resonance of the iron-containing protein B2 from ribonucleotide reductase. J Biol Chem.

[15] Stubbe J, Riggs-Gelasco P (1998). Harnessing free radicals: formation and function of the tyrosyl radical in ribonucleotide reductase. Trends Biochem Sci.

[16] Cotruvo JA, Stich TA, Britt RD, Stubbe J (2013). Mechanism of assembly of the dimanganese-tyrosyl radical cofactor of class Ib ribonucleotide reductase: enzymatic generation of superoxide is required for tyrosine oxidation via a Mn(III)Mn(IV) intermediate. J Am Chem Soc.

[17] Jiang W, Yun D, Saleh L (2007). A manganese(IV)/iron(III) cofactor in *Chlamydia trachomatis* ribonucleotide reductase. Science.

[18] Högbom M, Stenmark P, Voevodskaya N (2004). The radical site in chlamydial ribonucleotide reductase defines a new R2 subclass. Science.

[19] Voevodskaya N, Lendzian F, Ehrenberg A, Gräslund A (2007). High catalytic activity achieved with a mixed manganese–iron site in protein R2 of *Chlamydia* ribonucleotide reductase. FEBS Lett.

[20] Rose HR, Ghosh MK, Maggiolo AO (2018). Structural basis for superoxide activation of *Flavobacterium johnsoniae* class I ribonucleotide reductase and for radical initiation by its dimanganese cofactor. Biochemistry.

[21] Rozman Grinberg I, Berglund S, Hasan M (2019). Class Id ribonucleotide reductase utilizes a Mn_2_(IV, III) cofactor and undergoes large conformational changes on metal loading. J Biol Inorg Chem.

[22] Andersson CS, Högbom M (2009). A *Mycobacterium tuberculosis* ligand-binding Mn/Fe protein reveals a new cofactor in a remodeled R2-protein scaffold. Proc Natl Acad Sci USA.

[23] Griese JJ, Kositzki R, Schrapers P (2015). Structural basis for oxygen activation at a heterodinuclear manganese/iron cofactor. J Biol Chem.

[24] Roos K, Siegbahn PEM (2011). Oxygen cleavage with manganese and iron in ribonucleotide reductase from *Chlamydia trachomatis*. J Biol Inorg Chem.

[25] Martinie RJ, Blaesi EJ, Krebs C (2017). Evidence for a Di-μ-oxo diamond core in the Mn(IV)/Fe(IV) activation intermediate of ribonucleotide reductase from *Chlamydia trachomatis*. J Am Chem Soc.

[26] Miller EK, Trivelas NE, Maugeri PT (2017). Time-resolved investigations of heterobimetallic cofactor assembly in R2lox reveal distinct Mn/Fe intermediates. Biochemistry.

[27] Shafaat HS, Griese JJ, Pantazis DA (2014). Electronic structural flexibility of heterobimetallic Mn/Fe cofactors: R2lox and R2c proteins. J Am Chem Soc.

[28] Jiang W, Hoffart LM, Krebs C, Bollinger JM (2007). A manganese(IV)/iron(IV) intermediate in assembly of the manganese(IV)/iron(III) cofactor of *Chlamydia trachomatis* ribonucleotide reductase. Biochemistry.

[29] Kutin Y, Kositzki R, Branca RMM (2019). Chemical flexibility of heterobimetallic Mn/Fe cofactors: R2lox and R2c proteins. J Biol Chem.

[30] Kutin Y, Srinivas V, Fritz M (2016). Divergent assembly mechanisms of the manganese/iron cofactors in R2lox and R2c proteins. J Inorg Biochem.

[31] Cotruvo JA, Stubbe J (2010). An active dimanganese(III)–tyrosyl radical cofactor in *Escherichia coli* class Ib ribonucleotide reductase. Biochemistry.

[32] Cox N, Ogata H, Stolle P (2010). A tyrosyl−dimanganese coupled spin system is the native metalloradical cofactor of the R2F subunit of the ribonucleotide reductase of *Corynebacterium ammoniagenes*. J Am Chem Soc.

[33] Cotruvo JA, Stubbe J (2011). *Escherichia coli* class Ib ribonucleotide reductase contains a dimanganese(III)–tyrosyl radical cofactor in vivo. Biochemistry.

[34] Zhang Y, Stubbe J (2011). *Bacillus subtilis* class Ib ribonucleotide reductase is a dimanganese(III)–tyrosyl radical enzyme. Biochemistry.

[35] Martin JE, Imlay JA (2011). The alternative aerobic ribonucleotide reductase of *Escherichia coli*, NrdEF, is a manganese-dependent enzyme that enables cell replication during periods of iron starvation. Mol Microbiol.

[36] Högbom M (2010). The manganese/iron–carboxylate proteins: what is what, where are they, and what can the sequences tell us?. J Biol Inorg Chem.

[37] Griese JJ, Högbom M, IUCr (2019). Location-specific quantification of protein-bound metal ions by X-ray anomalous dispersion: Q-XAD. Acta Crystallogr Sect D Struct Biol.

[38] Torrents E, Sahlin M, Biglino D (2005). Efficient growth inhibition of *Bacillus anthracis* by knocking out the ribonucleotide reductase tyrosyl radical. Proc Natl Acad Sci USA.

[39] Grāve K, Lambert W, Berggren G (2019). Redox-induced structural changes in the di-iron and di-manganese forms of *Bacillus anthracis* ribonucleotide reductase subunit NrdF suggest a mechanism for gating of radical access. J Biol Inorg Chem.

[40] Klockenkämper R (1997). Total-reflection X-ray fluorescence analysis.

[41] Kabsch W (2010). XDS. Acta Crystallogr Sect D Biol Crystallogr.

[42] Winter G, Waterman DG, Parkhurst JM (2018). DIALS: implementation and evaluation of a new integration package. Acta Crystallogr Sect D Struct Biol.

[43] Evans PR, Murshudov GN (2013). How good are my data and what is the resolution?. Acta Crystallogr D Biol Crystallogr.

[44] McCoy AJ, Grosse-Kunstleve RW, Adams PD (2007). Phaser crystallographic software. J Appl Crystallogr.

[45] Adams PD, Afonine PV, Bunkóczi G (2010). PHENIX: a comprehensive python-based system for macromolecular structure solution. Acta Crystallogr D Biol Crystallogr.

[46] Emsley P, Lohkamp B, Scott WG, Cowtan K (2010). Features and development of Coot. Acta Crystallogr D Biol Crystallogr.

[47] Afonine PV, Grosse-Kunstleve RW, Echols N (2012). Towards automated crystallographic structure refinement with phenix.refine. Acta Crystallogr D Biol Crystallogr.

[48] Williams CJ, Headd JJ, Moriarty NW (2018). MolProbity: more and better reference data for improved all-atom structure validation. Protein Sci.

[49] The PyMOL molecular graphics system, version 2.0.0 Schrödinger, LLC. https://www.pymol.org/

[50] Kleywegt GJ, Jones TA (1996). xdlMAPMAN and xdlDATAMAN—programs for reformatting, analysis and manipulation of biomacromolecular electron-density maps and reflection data sets. Acta Crystallogr D Biol Crystallogr.

[51] Boal AK, Cotruvo JA, Stubbe J, Rosenzweig AC (2010). Structural basis for activation of class Ib ribonucleotide reductase. Science.

