# Supplementary material for: The Bacillus anthracis class Ib ribonucleotide reductase subunit NrdF intrinsically selects manganese over iron
Source: J Biol Inorg Chem. 2020 Apr 15;25(4):571–82. doi: 10.1007/s00775-020-01782-3 (PMC7239806; doi:10.1007/s00775-020-01782-3)
Supplement: Supplementary file 1 — Supplementary file1 (PDF 9805 kb) [file 775_2020_1782_MOESM1_ESM.pdf]

Supplementary information

The *Bacillus anthracis* class Ib ribonucleotide reductase subunit NrdF intrinsically selects manganese over iron

Kristīne Grāve[1], Julia J. Griese[1,2], Gustav Berggren[3], Matthew D. Bennett[1] and Martin Högbom✉[1]

1. Department of Biochemistry and Biophysics, Stockholm University. Svante Arrhenius väg 16C, SE-10691, Stockholm, Sweden.
2. Department of Cell and Molecular Biology, Uppsala University. BMC, Box 596, SE-75124, Uppsala, Sweden.
3. Department of Chemistry, Ångström Laboratory, Uppsala University. Lägerhyddsvägen 1, SE-75120, Uppsala, Sweden.

✉ Corresponding author: hogbom@dbb.su.se

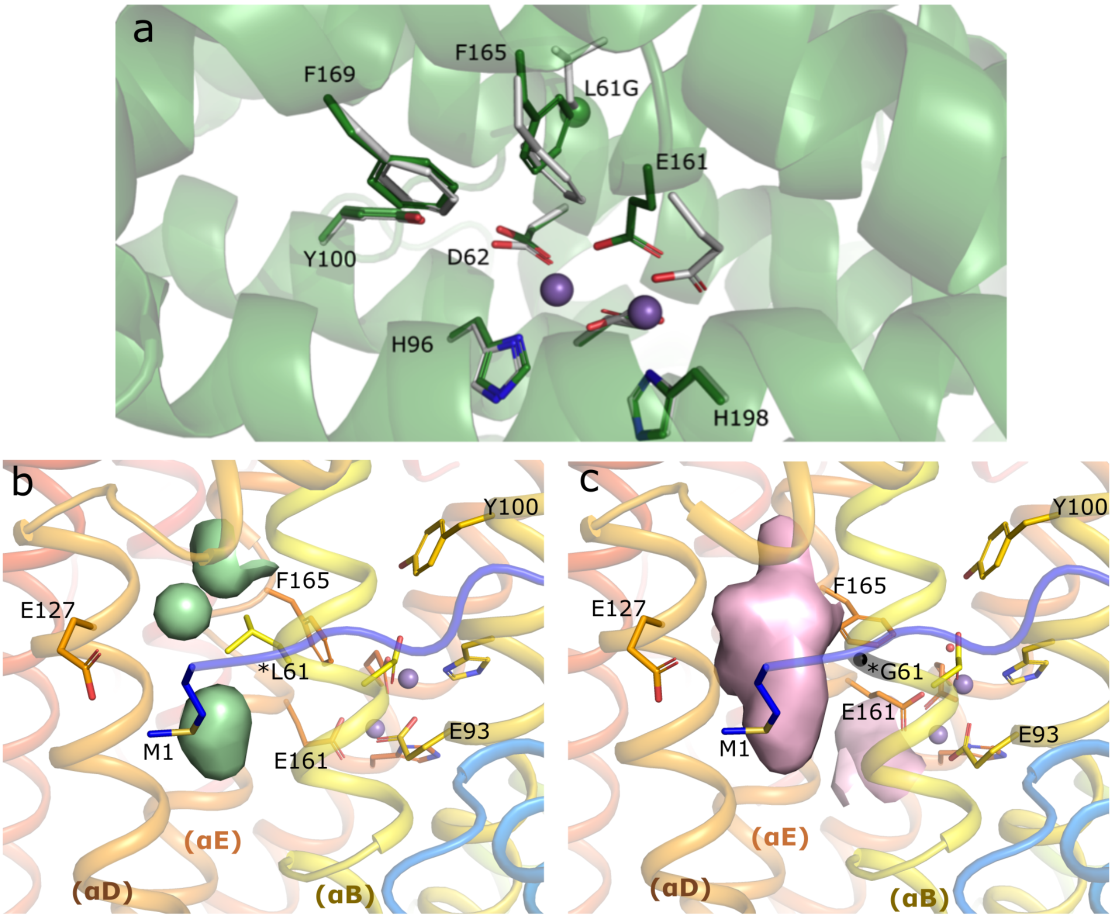

Supplementary Figure 1. (a) *Stick representation of side chain rearrangements in the vicinity of the metal site in L61G mutant BaR2b protein (green) compared to wild-type protein (white). Gly61 is shown as a green sphere. Mn ions are shown as purple spheres. Solvent and Glu195 are hidden for clarity.* ***(b)*** *Surface-connected pocket (green surface) in wild type BaR2b gated by Met1 and Glu127 residues. Leucine residue subjected to mutation is indicated with an asterisk. Protein is colored in rainbow (blue – N-terminus, red – C-terminus).* ***(c)*** *Surface-connected pocket (pink surface) in L61G variant BaR2b gated by Met1 and Glu127 residues. Gly61 side chain is shown as a black sphere and indicated with an asterisk. Protein is colored in rainbow (blue – N-terminus, red – C-terminus).*

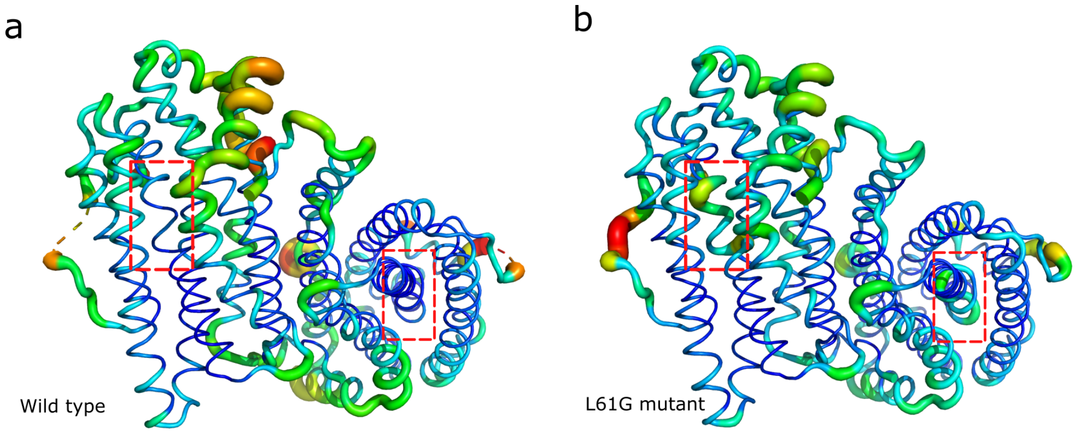

Supplementary Figure 2. *B-factors of Cα-atoms in the* ***(a)*** *wild-type and* ***(b)*** *L61G mutant BaR2b. B-factors are indicated by the color range from dark blue (lowest B factor) to red (highest B factor) and the thickness of the ribbon. The regions of* $\pi/3_{10}$ *helix of αE (residues 159 – 168) are indicated with a red rectangle.*

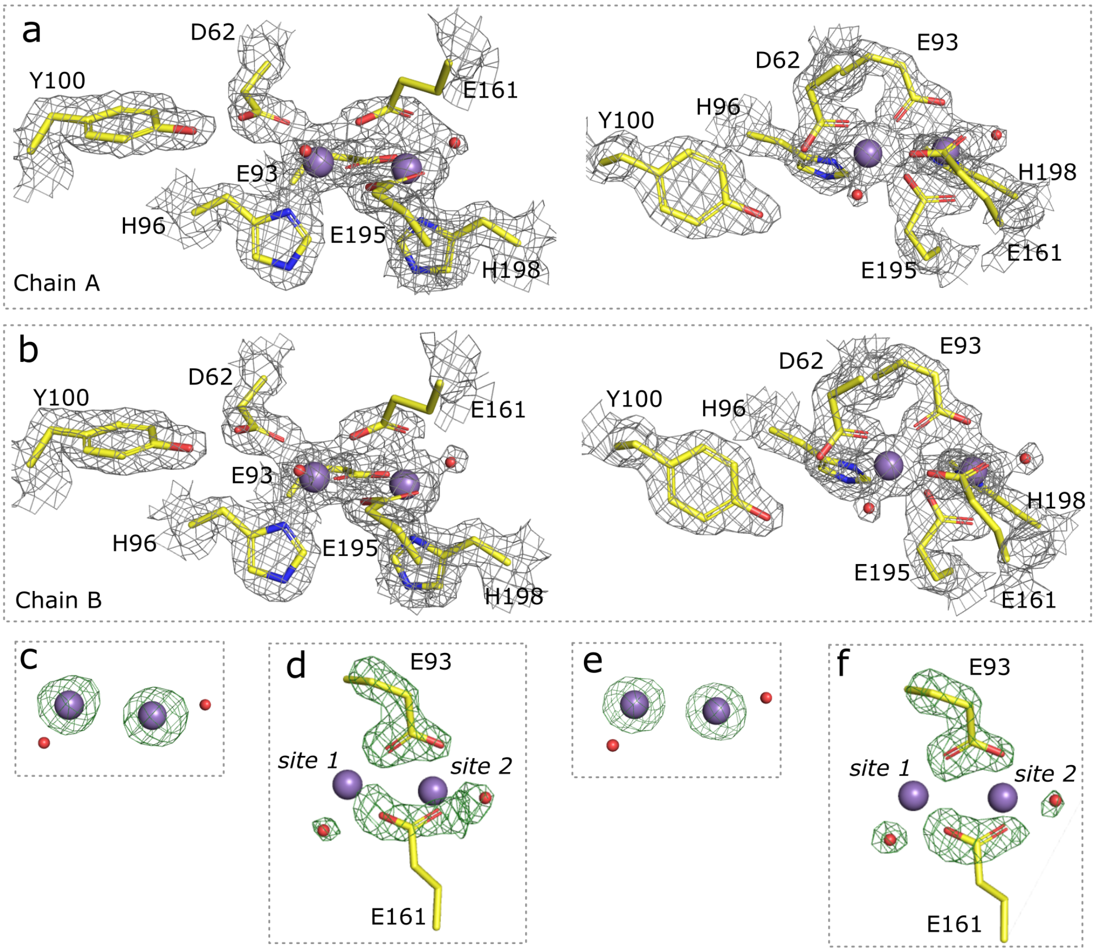

Supplementary Figure 3*. Electron density map for the metal sites in L61G variant BaR2b crystals anaerobically reconstituted with equal amounts on* Mn^{II} *and* Fe^{II}*.* ***(a) Left****: selected view on the metal site in chain A with a focus on E161.* ***Right****: selected view on the metal site in chain A with a focus on metal-coordinating solvent.* ***(b) Left****: selected view on the metal site in chain B with a focus on E161.* ***Right****: selected view on the metal site in chain B with a focus on metal-coordinating solvent.* ***(c)*** *Omit map (green mesh) for the metal ions in chain A is contoured at 7σ and shown within 2.2 Å from the metal ions.* ***(d)*** *Omit map (green mesh) for metal-coordinating solvent, E93 and E161 residues in protein chain A is contoured at 6σ and shown within 2 Å.* ***(e)*** *Omit map (green mesh) for the metal ions in chain B is contoured at 7σ and shown within 2.2 Å from the metal ions.* ***(f)*** *Omit map (green mesh) for metal-coordinating solvent, E93 and E161 residues in protein chain B is contoured at 6σ and shown within 2 Å.*

$2mF_o$-DF_c electron density (grey mesh) is contoured at 1.5σ.

Manganese ions are shown as purple spheres, solvent – as red spheres.

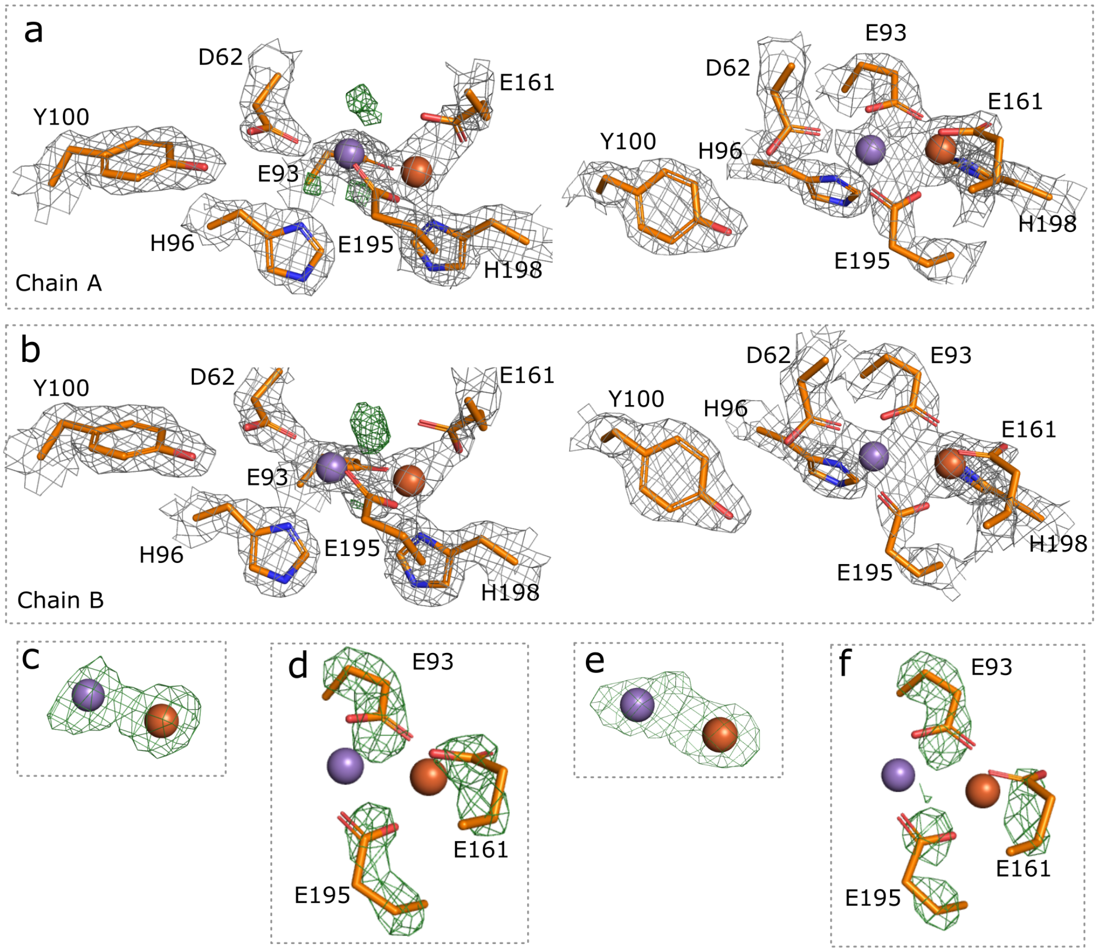

***Supplementary Figure 4**. Electron density map for the metal sites in L61G variant BaR2b crystals aerobically reconstituted with equal amounts on Mn^{II} and Fe^{II}. **(a) Left:** selected view on the metal site in chain A with a focus on E161. mF_o-DF_c positive difference density around the metal site (green) is contoured at 3.5σ and shown within 3 Å; **Right:** selected view on the metal site in chain A with a focus on E93 and E195 **(b) Left:** selected view on the metal site in chain B with a focus on E161. mF_o-DF_c positive difference density around the metal site (green) is contoured at 3.5σ and shown within 3 Å; **Right**: selected view on the metal site in chain B with a focus on E93 and E195 **(c)** Omit map (green mesh) for the metal ions in chain A is contoured at 5σ and shown within 3 Å from the metal ions. **(d)** Omit map (green mesh) for selected residues in protein chain A is contoured at 5σ and shown within 3 Å. **(e)** Omit map (green mesh) for the metal ions in chain B is contoured at 5σ and shown within 3 Å from the metal ions. **(f)** Omit map (green mesh) for selected residues in protein chain B is contoured at 5σ and shown within 3 Å.*

$2mF_o$-DF_c electron density (grey mesh) is contoured at 1.5σ.

Manganese ions are shown as purple spheres, iron – as orange spheres.

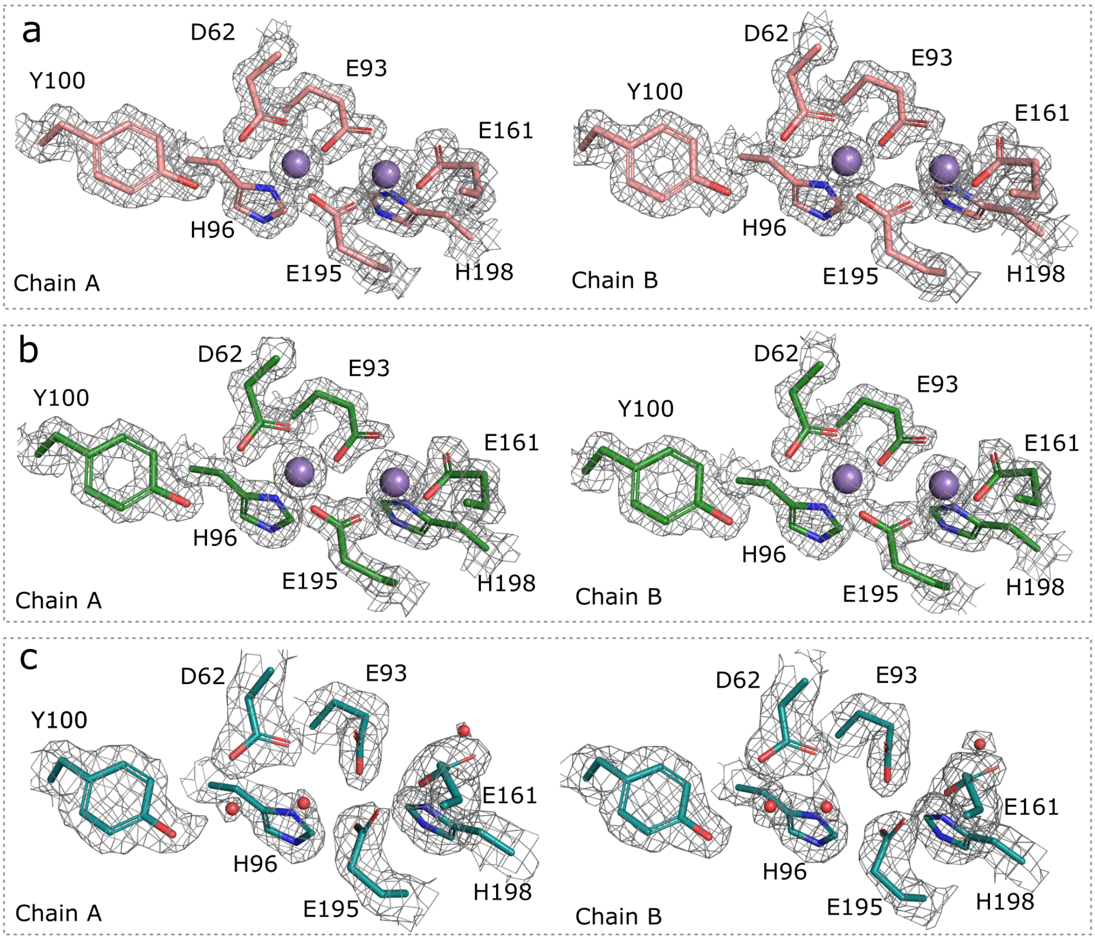

Supplementary Figure 5*. Electron density map for the metal sites in wild-type and L61G variant apo BaR2b crystals.* ***(a)*** *$2mF_o$-DF_c electron density (contoured at 2σ) for wild-type protein crystal soaked with equal amounts on Mn^{II} and Fe^{II} in presence of oxygen.* ***(b)*** *$2mF_o$-DF_c electron density (contoured at 2σ) for wild-type protein crystal anaerobically soaked with equal amounts on Mn^{II} and Fe^{II}.* ***(c)*** *$2mF_o$-DF_c electron density (contoured at 1.3σ) for L61G variant metal-free BaR2b crystal.*

Structure and electron density for the metal site in chain A of each crystal is shown on the ***left*** *and in chain B – on the* ***right*** *side of each panel.*

Manganese ions are shown as purple spheres, solvent - as red spheres.

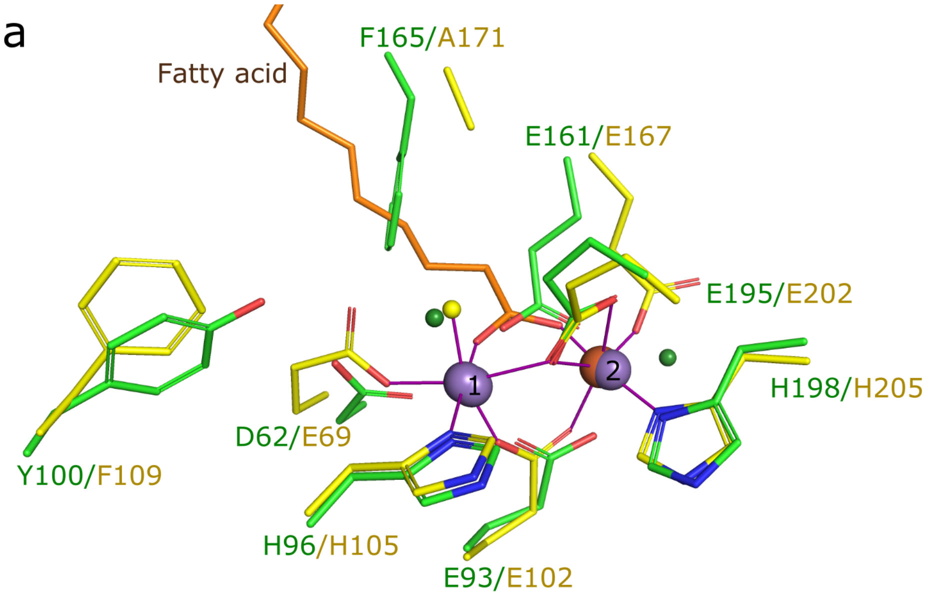

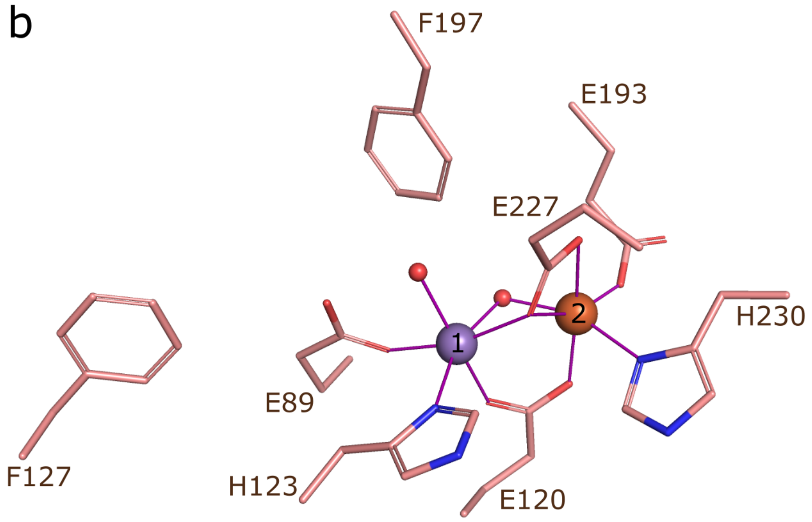

Supplementary Figure 6. *Structural comparison of* Bacillus anthracis *L61G variant,* Geobacillus kaustophilus *R2lox* [1] *(PDB: 4HR4) and* Chlamydia trachomatis *R2c* [2] *(PDB: 4M1I) metal sites.* ***(a)*** *Alignment of the L61G variant* B. anthracis *R2b (green) and* G. kaustophilus *R2lox (yellow) reconstituted with metal ions showing similarities in metal coordination by carboxylate ligands. E161 in* B. anthracis *R2b coordinates metal ions in a similar fashion to the fatty acid in R2lox. Solvent molecule coordinating metal ion in site 2 in* B. anthracis *R2 is in equivalent position of monodentate chelating E202 in R2lox.* ***(b)*** *Equivalent view of* C. trachomatis *R2c metal site.*

Supplementary Table 1. Relative amounts of Mn and Fe in the two metal sites obtained when reconstituting the cofactor in the absence or presence of oxygen. Each replicate represents one metal binding site in *Ba*R2b protein dimer. This data was used for the construction of Figure 4.

	Anoxic conditions Wild type				Aerobic conditions Wild type			
	Site 1		Site 2		Site 1		Site 2	
Replicate	Mn	Fe	Mn	Fe	Mn	Fe	Mn	Fe
1	0.86	0.14	0.81	0.19	0.86	0.14	0.68	0.32
2	0.83	0.17	0.73	0.27	0.86	0.14	0.71	0.29
3	0.76	0.24	0.87	0.13	0.74	0.26	0.73	0.27
4	0.69	0.31	0.79	0.21	0.73	0.27	0.69	0.31
5	0.73	0.27	0.63	0.37	0.84	0.16	0.86	0.14
6	0.81	0.19	0.66	0.34	0.96	0.04	0.89	0.11

	Anoxic conditions L61G variant				Aerobic conditions L61G variant			
	Site 1		Site 2		Site 1		Site 2	
Replicate	Mn	Fe	Mn	Fe	Mn	Fe	Mn	Fe
1	0.64	0.36	0.65	0.35	0.90	0.10	0	1.00
2	0.65	0.35	0.68	0.32	0.91	0.09	0	1.00
3	0.76	0.24	0.73	0.27	0.47	0.53	0	1.00
4	0.88	0.12	0.67	0.33	0.49	0.51	0	1.00
5	0.59	0.41	0.52	0.48	-	-	-	-
6	0.66	0.34	0.45	0.55	-	-	-	-

Supplementary Table 2. Anomalous data collection statistics.

Crystal	†Aerobic – 1 (WT)	†Aerobic – 2 (WT)	†Aerobic – 3 (WT)
Dataset	Mn peak	Mn peak	Mn peak
Wavelength (Å)	1.89	1.89	1.89
Energy (eV)	6559	6559	6559
Resolution range (Å)	46.0 – 3.0 (3.2 – 3.0)	46.0 – 3.0 (3.2 – 3.0)	46.0 – 3.0 (3.2 – 3.0)
Space group	$P2_1$	$P2_1$	$P2_1$
Cell dimensions			
a, *b*, *c* (Å)	57.3, 60.7, 95.9	57.1, 60.2, 95.9	57.2, 60.5, 95.8
Unique angle: β (°)	106.5	106.5	106.5
Total reflections	81636 (12852)	80871 (12774)	81779 (13125)
Unique reflections	12592 (2012)	12470 (2003)	12523 (2131)
Multiplicity	6.5 (6.4)	6.5 (6.4)	6.5 (6.5)
Completeness (%)	98.1 (97.7)	98.3 (98.3)	98.2 (98.7)
Mean I/sigma (I)	31.1 (27.3)	32.4 (28.0)	31.0 (28.3)
*R_{merge}	0.04 (0.05)	0.03 (0.05)	0.04 (0.05)
*R_{meas}	0.05 (0.06)	0.04 (0.06)	0.05 (0.06)
*R_{pim}	0.02 (0.03)	0.02 (0.03)	0.03 (0.03)
$CC_{1/2}$	0.99 (0.99)	0.99 (0.99)	0.99 (0.99)
Anomalous completeness (%)	96.5 (96.1)	96.8 (96.6)	97.2 (97.1)
Anomalous multiplicity	3.3 (3.3)	3.3 (3.3)	3.3 (3.3)
#Anomalous signal	0.87 (0.75)	0.85 (0.72)	0.86 (0.77)

Values in parentheses are for the highest resolution shell.

†Apoprotein crystals were soaked with Mn(II) and Fe(II) for 20 – 30 minutes in oxygen-containing buffer.

§ Apoprotein crystals were soaked with Mn(II) and Fe(II) for 45 – 60 minutes in reducing anoxic conditions.

* These values are within I+/I- from the AIMLESS log file

#These values are from the XSCALE log file (=SigAno, mean anomalous difference in units of its estimated standard deviation (|F(+)-F(-)|/Sigma). F(+), F(-) are structure factor estimates obtained from the merged intensity observations in each parity class).

Supplementary Table 2 (continued). Anomalous data collection statistics.

Crystal	†Aerobic – 1 (WT)	†Aerobic – 2 (WT)	†Aerobic – 3 (WT)
Dataset	Fe peak	Fe peak	Fe peak
Wavelength (Å)	1.74	1.74	1.74
Energy (eV)	7132	7132	7132
Resolution range (Å)	46.0 – 3.0	46.0 – 3.0	46.0 – 3.0
	(3.2 – 3.0)	(3.2 – 3.0)	(3.2 – 3.0)
Space group	$P2_1$	$P2_1$	$P2_1$
Cell dimensions			
a, *b*, *c* (Å)	57.4, 60.7, 95.9	57.2, 60.3, 95.9	57.2, 60.6, 95.9
Unique angle: β (°)	106.5	106.5	106.5
Total reflections	82082 (13296)	81098 (13180)	82316 (13335)
Unique reflections	12626 (2030)	12517 (2024)	12592 (2029)
Multiplicity	6.5 (6.5)	6.5 (6.5)	6.5 (6.6)
Completeness (%)	98.3 (98.5)	98.2 (98.7)	98.5 (99.1)
Mean I/sigma (I)	32.3 (28.7)	40.5 (34.7)	39.3 (35.1)
*R_{merge}	0.04 (0.05)	0.03 (0.04)	0.03 (0.04)
*R_{meas}	0.05 (0.06)	0.03 (0.05)	0.04 (0.05)
*R_{pim}	0.02 (0.03)	0.02 (0.03)	0.02 (0.03)
$CC_{1/2}$	0.99 (0.99)	1.00 (0.99)	0.99 (0.99)
Anomalous completeness (%)	97.0 (97.2)	96.9 (97.2)	97.5 (97.9)
Anomalous multiplicity	3.3 (3.3)	3.3 (3.3)	3.3 (3.4)
#Anomalous signal	0.80 (0.67)	0.92 (0.75)	0.96 (0.82)

Values in parentheses are for the highest resolution shell.

†Apoprotein crystals were soaked with Mn(II) and Fe(II) for 20 – 30 minutes in oxygen-containing buffer.

§ Apoprotein crystals were soaked with Mn(II) and Fe(II) for 45 – 60 minutes in reducing anoxic conditions.

* These values are within I+/I- from the AIMLESS log file

#These values are from the XSCALE log file (=SigAno, mean anomalous difference in units of its estimated standard deviation (|F(+)-F(-)|/Sigma). F(+), F(-) are structure factor estimates obtained from the merged intensity observations in each parity class).

Supplementary Table 2 (continued). Anomalous data collection statistics.

Crystal	§Reduced – 1 (WT)	§Reduced – 2 (WT)	§Reduced – 3 (WT)
Dataset	Mn peak	Mn peak	Mn peak
Wavelength (Å)	1.89	1.89	1.89
Energy (eV)	6559	6559	6559
Resolution range (Å)	46.0 – 3.0 (3.2 – 3.0)	46.0 – 3.0 (3.2 – 3.0)	46.0 – 3.0 (3.2 – 3.0)
Space group	$P2_1$	$P2_1$	$P2_1$
Cell dimensions			
a, *b*, *c* (Å)	57.4, 61.0, 95.4	57.4, 61.1, 95.5	57.4, 61.1, 95.5
Unique angle: β (°)	106.6	106.6	106.6
Total reflections	82374 (13018)	82888 (13144)	82716 (13101)
Unique reflections	12515 (1990)	12244 (1955)	12630 (2029)
Multiplicity	6.6 (6.5)	6.8 (6.7)	6.5 (6.5)
Completeness (%)	97.7 (97.2)	95.5 (95.3)	98.1 (98.5)
Mean I/sigma (I)	36.5 (34.0)	38.8 (33.4)	30.7 (24.2)
*R_{merge}	0.03 (0.05)	0.04 (0.04)	0.04 (0.06)
*R_{meas}	0.04 (0.05)	0.04 (0.05)	0.05 (0.07)
*R_{pim}	0.02 (0.03)	0.02 (0.03)	0.03 (0.04)
$CC_{1/2}$	0.99 (0.99)	0.99 (0.99)	0.99 (0.99)
Anomalous completeness (%)	96.3 (95.5)	94.9 (94.4)	96.8 (95.5)
Anomalous multiplicity	3.3 (3.4)	3.4 (3.4)	3.3 (3.3)
#Anomalous signal	1.03 (1.06)	1.07 (1.09)	0.88 (0.83)

Values in parentheses are for the highest resolution shell.

†Apoprotein crystals were soaked with Mn(II) and Fe(II) for 20 – 30 minutes in oxygen-containing buffer.

§ Apoprotein crystals were soaked with Mn(II) and Fe(II) for 45 – 60 minutes in reducing anoxic conditions.

* These values are within I+/I- from the AIMLESS log file

#These values are from the XSCALE log file (=SigAno, mean anomalous difference in units of its estimated standard deviation (|F(+)-F(-)|/Sigma). F(+), F(-) are structure factor estimates obtained from the merged intensity observations in each parity class).

Supplementary Table 2 (continued). Anomalous data collection statistics.

Crystal	§Reduced – 1 (WT)	§Reduced – 2 (WT)	§Reduced – 3 (WT)
Dataset	Fe peak	Fe peak	Fe peak
Wavelength (Å)	1.74	1.74	1.74
Energy (eV)	7132	7132	7132
Resolution range (Å)	46.0 – 3.0	46.0 – 3.0	46.0 – 3.0
Space group	P21	P21	P21
Cell dimensions			
a, *b*, *c* (Å)	57.4, 61.0, 95.4	57.4, 61.0, 95.5	57.5, 61.1, 95.5
Unique angle: β (°)	106.6	106.6	106.6
Total reflections	81872 (13127)	83676 (13546)	83015 (13429)
Unique reflections	12493 (1994)	12299 (1981)	12686 (2044)
Multiplicity	6.6 (6.6)	6.8 (6.8)	6.5 (6.6)
Completeness (%)	97.4 (97.1)	95.9 (96.3)	98.5 (99.0)
Mean I/sigma (I)	40.4 (36.8)	42.7 (36.6)	44.7 (39.0)
*Rmerge	0.03 (0.04)	0.03 (0.04)	0.03 (0.04)
*Rmeas	0.04 (0.05)	0.04 (0.05)	0.03 (0.04)
*Rpim	0.02 (0.03)	0.02 (0.02)	0.02 (0.02)
CC1/2	0.99 (0.99)	0.99 (0.99)	0.99 (0.99)
Anomalous completeness (%)	96.1 (95.5)	95.3 (95.3)	97.1 (96.8)
Anomalous multiplicity	3.3 (3.4)	3.5 (3.5)	3.3 (3.4)
#Anomalous signal	0.93 (0.89)	0.95 (0.89)	0.97 (0.91)

Values in parentheses are for the highest resolution shell.

†Apoprotein crystals were soaked with Mn(II) and Fe(II) for 20 – 30 minutes in oxygen-containing buffer.

§ Apoprotein crystals were soaked with Mn(II) and Fe(II) for 45 – 60 minutes in reducing anoxic conditions.

* These values are within I+/I- from the AIMLESS log file

#These values are from the XSCALE log file (=SigAno, mean anomalous difference in units of its estimated standard deviation (|F(+)-F(-)|/Sigma). F(+), F(-) are structure factor estimates obtained from the merged intensity observations in each parity class).

Supplementary Table 2 (continued). Anomalous data collection statistics.

Crystal	†Aerobic – 1 (L61G)	†Aerobic – 2 (L61G)
Dataset	Mn peak	Mn peak
Wavelength (Å)	1.89	1.89
Energy (eV)	6589	6589
Resolution range (Å)	46.0 – 3.0 (3.2 – 3.0)	46.0 – 3.0 (3.2 – 3.0)
Space group	$P2_1$	$P2_1$
Cell dimensions		
a, *b*, *c* (Å)	57.2, 60.3, 95.9	56.9, 60.1, 95.9
Unique angle: β (°)	106.5	106.6
Total reflections	81032 (13044)	80262 (12854)
Unique reflections	12546 (2011)	12336 (1974)
Multiplicity	6.5 (6.5)	6.5 (6.5)
Completeness (%)	98.6 (98.3)	98.0 (98.0)
Mean I/sigma (I)	15.6 (12.08)	13.5 (7.9)
*R_{merge}	0.07 (0.09)	0.08 (0.17)
*R_{meas}	0.09 (0.11)	0.10 (0.20)
*R_{pim}	0.04 (0.04)	0.05 (0.11)
$CC_{1/2}$	0.99 (0.99)	0.99 (0.98)
Anomalous completeness (%)	97.0 (97.3)	96.6 (97.2)
Anomalous multiplicity	3.3 (3.3)	3.3 (3.4)
#Anomalous signal	0.68 (0.51)	0.77 (0.65)

Values in parentheses are for the highest resolution shell.

†Apoprotein crystals were soaked with Mn(II) and Fe(II) for 20 – 30 minutes in oxygen-containing buffer.

§ Apoprotein crystals were soaked with Mn(II) and Fe(II) for 45 – 60 minutes in reducing anoxic conditions.

* These values are within I+/I- from the AIMLESS log file

#These values are from the XSCALE log file (=SigAno, mean anomalous difference in units of its estimated standard deviation (|F(+)-F(-)|/Sigma). F(+), F(-) are structure factor estimates obtained from the merged intensity observations in each parity class)

Supplementary Table 2 (continued). Anomalous data collection statistics.

Crystal	†Aerobic – 1 (L61G)	†Aerobic – 2 (L61G)
Dataset	Fe peak	Fe peak
Wavelength (Å)	1.74	1.74
Energy (eV)	7162	7162
Resolution range (Å)	46.0 – 3.0 (3.2 – 3.0)	46.0 – 3.0 (3.2 – 3.0)
Space group	$P2_1$	$P2_1$
Cell dimensions		
a, *b*, *c* (Å)	57.2, 60.3, 95.9	56.9, 60.1, 95.9
Unique angle: β (°)	106.5	106.6
Total reflections	81565 (13218)	80740 (12997)
Unique reflections	12545 (2017)	12384 (1981)
Multiplicity	6.5 (6.6)	6.5 (6.6)
Completeness (%)	98.6 (98.6)	98.4 (98.6)
Mean I/sigma (I)	15.9 (13.05)	14.2 (8.8)
*R_{merge}	0.07 (0.09)	0.08 (0.14)
*R_{meas}	0.09 (0.10)	0.09 (0.17)
*R_{pim}	0.05 (0.06)	0.04 (0.06)
$CC_{1/2}$	0.99 (0.99)	0.99 (0.99)
Anomalous completeness (%)	96.9 (97.9)	96.8 (97.4)
Anomalous multiplicity	3.3 (3.4)	3.3 (3.4)
#Anomalous signal	0.65 (0.48)	0.74 (0.64)

Values in parentheses are for the highest resolution shell.

†Apoprotein crystals were soaked with Mn(II) and Fe(II) for 20 – 30 minutes in oxygen-containing buffer.

§ Apoprotein crystals were soaked with Mn(II) and Fe(II) for 45 – 60 minutes in reducing anoxic conditions.

* These values are within I+/I- from the AIMLESS log file

#These values are from the XSCALE log file (=SigAno, mean anomalous difference in units of its estimated standard deviation (|F(+)-F(-)|/Sigma). F(+), F(-) are structure factor estimates obtained from the merged intensity observations in each parity class).

Supplementary Table 2 (continued). Anomalous data collection statistics.

Crystal	§Reduced – 1 (L61G)	§Reduced – 2 (L61G)	§Reduced – 3 (L61G)
Dataset	Mn peak	Mn peak	Mn peak
Wavelength (Å)	1.89	1.89	1.89
Energy (eV)	6589	6589	6589
Resolution range (Å)	46.0 – 3.0 (3.2 – 3.0)	46.0 – 3.0 (3.2 – 3.0)	46.0 – 3.0 (3.2 – 3.0)
Space group	$P2_1$	$P2_1$	$P2_1$
Cell dimensions			
a, *b*, *c* (Å)	57.2, 60.4, 95.9	57.2, 60.5, 96.0	57.2, 60.3, 95.9
Unique angle: β (°)	106.6	106.7	106.5
Total reflections	80270 (11993)	80141 (12976)	79982 (12978)
Unique reflections	12547 (2008)	12553 (2014)	12503 (2018)
Multiplicity	6.4 (6.0)	6.4 (6.4)	6.4 (6.4)
Completeness (%)	98.5 (97.5)	98.3 (98.1)	98.3 (98.4)
Mean I/sigma (I)	23.9 (18.7)	17.7 (11.8)	21.4 (12.5)
*R_{merge}	0.05 (0.05)	0.06 (0.09)	0.05 (0.10)
*R_{meas}	0.5 (0.06)	0.07 (0.11)	0.06 (0.11)
*R_{pim}	0.03 (0.03)	0.04 (0.06)	0.03 (0.06)
$CC_{1/2}$	0.99 (0.99)	0.99 (0.99)	0.99 (0.99)
Anomalous completeness (%)	96.7 (95.7)	96.4 (96.5)	96.6 (97.1)
Anomalous multiplicity	3.3 (3.1)	3.3 (3.3)	3.3 (3.3)
#Anomalous signal	0.66 (0.54)	0.62 (0.56)	0.79 (0.64)

Values in parentheses are for the highest resolution shell.

†Apoprotein crystals were soaked with Mn(II) and Fe(II) for 20 – 30 minutes in oxygen-containing buffer.

§ Apoprotein crystals were soaked with Mn(II) and Fe(II) for 45 – 60 minutes in reducing anoxic conditions.

* These values are within I+/I- from the AIMLESS log file

#These values are from the XSCALE log file (=SigAno, mean anomalous difference in units of its estimated standard deviation (|F(+)-F(-)|/Sigma). F(+), F(-) are structure factor estimates obtained from the merged intensity observations in each parity class)

Supplementary Table 2 (continued). Anomalous data collection statistics.

Crystal	§Reduced – 1 (L61G)	§Reduced – 2 (L61G)	§Reduced – 3 (L61G)
Dataset	Fe peak	Fe peak	Fe peak
Wavelength (Å)	1.74	1.74	1.74
Energy (eV)	7162	7162	7162
Resolution range (Å)	46.0 – 3.0 (3.2 – 3.0)	46.0 – 3.0 (3.2 – 3.0)	46.0 – 3.0 (3.2 – 3.0)
Space group	$P2_1$	$P2_1$	$P2_1$
Cell dimensions			
a, *b*, *c* (Å)	57.2, 60.4, 95.9	57.2, 60.5, 96.0	57.2, 60.3, 95.9
Unique angle: β (°)	106.6	106.7	106.5
Total reflections	81564 (13292)	79323 (13052)	80240 (13070)
Unique reflections	12573 (2043)	12527 (2012)	12527 (2028)
Multiplicity	6.5 (6.5)	6.3 (6.5)	6.4 (6.4)
Completeness (%)	98.7 (99.2)	98.1 (98.0)	98.4 (98.8)
Mean I/sigma (I)	19.9 (16.5)	17.5 (9.7)	24.17 (15.1)
*R_{merge}	0.04 (0.05)	0.07 (0.12)	0.04 (0.08)
*R_{meas}	0.05 (0.06)	0.08 (0.14)	0.05 (0.09)
*R_{pim}	0.03 (0.03)	0.04 (0.08)	0.03 (0.05)
$CC_{1/2}$	0.99 (0.99)	0.99 (0.99)	0.99 (0.99)
Anomalous completeness (%)	97.2 (98.0)	95.5 (96.4)	96.5 (97.6)
Anomalous multiplicity	3.3 (3.4)	3.2 (3.3)	3.3 (3.3)
#Anomalous signal	0.54 (0.43)	0.81 (0.63)	0.88 (0.65)

Values in parentheses are for the highest resolution shell.

†Apoprotein crystals were soaked with Mn(II) and Fe(II) for 20 – 30 minutes in oxygen-containing buffer.

§ Apoprotein crystals were soaked with Mn(II) and Fe(II) for 45 – 60 minutes in reducing anoxic conditions.

* These values are within I+/I- from the AIMLESS log file

#These values are from the XSCALE log file (=SigAno, mean anomalous difference in units of its estimated standard deviation (|F(+)-F(-)|/Sigma). F(+), F(-) are structure factor estimates obtained from the merged intensity observations in each parity class).

Supplementary References

1. Griese JJ, Roos K, Cox N, et al (2013) Direct observation of structurally encoded metal discrimination and ether bond formation in a heterodinuclear metalloprotein. *Proceedings of the National Academy of Sciences of the United States of America* **110**, 17189–17194. https://doi.org/10.1073/pnas.1304368110
2. Dassama LMK, Krebs C, Bollinger JM, et al (2013) Structural basis for assembly of the MnIV/FeIII cofactor in the class Ic ribonucleotide reductase from Chlamydia trachomatis. *Biochemistry* **52**, 6424–6436. https://doi.org/10.1021/bi400819x
